# Engineered Probiotic-Based Biomaterials for Inflammatory Bowel Disease Treatment

**DOI:** 10.7150/thno.103983

**Published:** 2025-02-18

**Authors:** Guangze Sang, Bingkai Wang, Yujie Xie, Yu Chen, Feng Yang

**Affiliations:** 1 Department of Inorganic Chemistry, School of Pharmacy, Naval Medical University, Shanghai, 200433, P. R. China.; 2 School of Medicine, Shanghai University, Shanghai, 200444, P. R. China.; 3 Materdicine lab, School of Life Sciences, Shanghai University, Shanghai, 200444, P. R. China.

**Keywords:** engineered probiotic-based materials, living materials, inflammatory bowel disease, probiotics

## Abstract

Inflammatory bowel disease (IBD) is a chronic condition affecting the intestines, marked by immune-mediated inflammation. This disease is known for its recurrent nature and the challenges it presents in treatment. Recently, probiotic have gained attention as a promising alternative to traditional small molecular drugs and monoclonal antibody chemotherapies for IBD. Probiotic, recognized as a “living” therapeutic agent, offers targeted treatment with minimal side effects and the flexibility for biological modifications, making them highly effective for IBD management. This comprehensive review presents the latest advancements in engineering probiotic-based materials, ranging from basic treatment mechanisms to the modification techniques used in IBD management. It delves deep into how probiotic produces therapeutic effects in the intestinal environment and discusses various strategies to enhance probiotic's efficacy, including genetic modifications and formulation improvements. Additionally, the review addresses the challenges, practical application conditions, and future research directions of probiotic-based therapies in IBD treatment, providing insights into their feasibility and potential clinical implications.

## 1. Introduction

**Figure 1:**
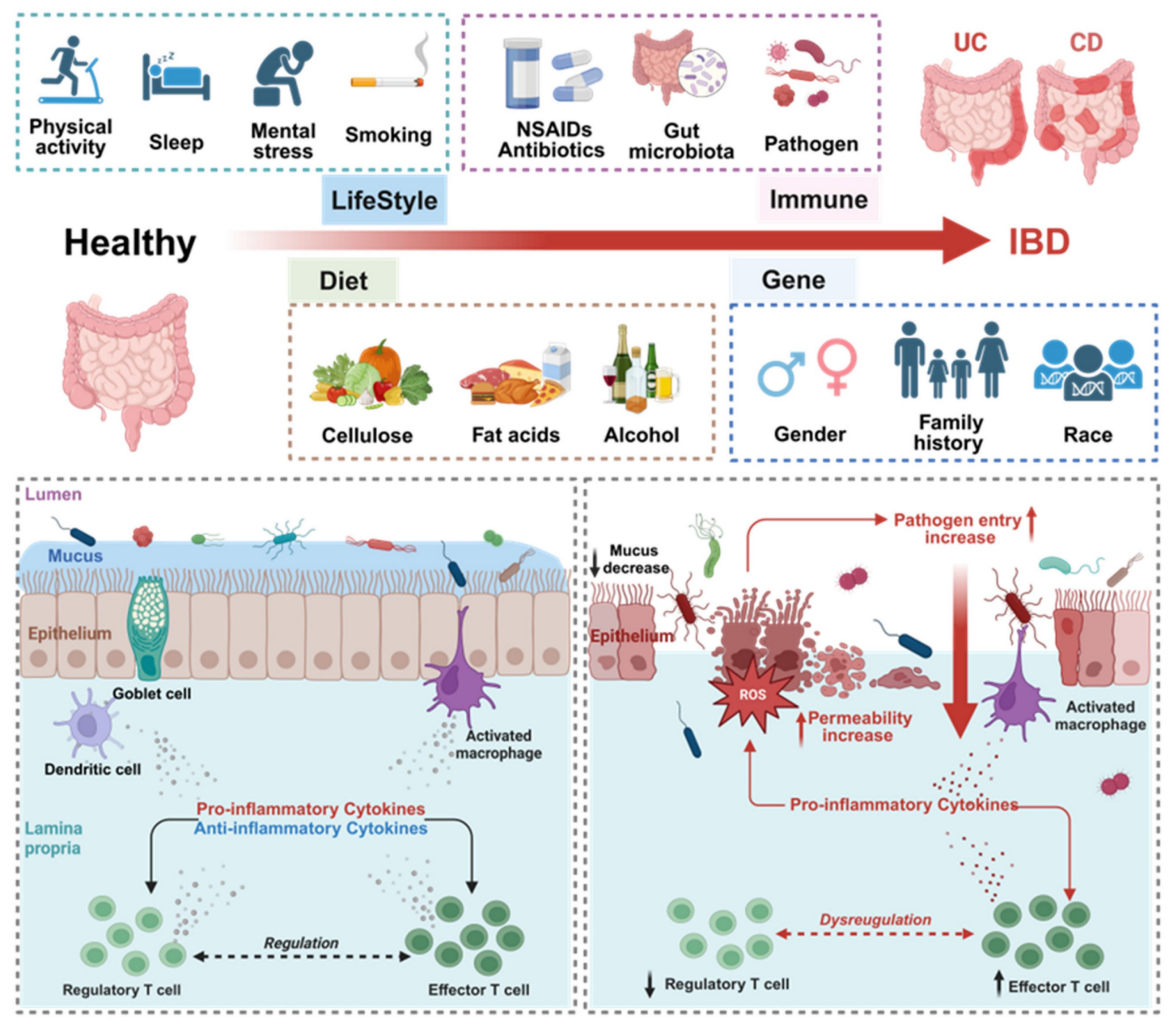
Comparison of intestinal homeostasis in healthy and IBD conditions and possible pathogenic factors. The current research results on the pathogenesis of IBD are still unclear, and it is generally believed to be related to individual lifestyle habits, immune status, dietary habits, and genetic background. In healthy organisms, the intestinal epithelium maintains a state of immune tolerance. However, upon exposure to inflammatory stimuli, the protective mucus layer and the epithelial cell barrier can become compromised. This leads to abnormal stimulation of immune cells by immunogenic substances, triggering a local immune response. If the antigens are not promptly eliminated, inflammation ensues, further damaging the intestinal barrier. This creates a vicious cycle, perpetuating long-term intestinal inflammation. Created with https://BioRender.com.

Inflammatory bowel disease (IBD) is an immune-mediated and chronic intestinal disorder, encompassing conditions such as Crohn's disease (CD) and ulcerative colitis (UC), both of which have similar clinical manifestations, mainly including abdominal pain, diarrhea, bloody stools, weight loss, anemia, and low-grade fever. Currently, the exact cause of IBD is still unclear, but it is commonly associated with a combination of lifestyle, behavioral habits, immune system functionality, and environmental and genetic factors (**Figure 1**) 1. The current mainstream pharmacological interventions of IBD treatment include non-steroidal anti-inflammatory drugs (5-aminosalicylic acid), corticosteroids (budesonide, prednisone and prednisolone), monoclonal antibodies (adalimumab, infliximab), Janus kinase inhibitors (tofacitinib, upadacitinib), immunosuppressants (methotrexate, cyclosporine, tacrolimus). As the emerging evidence highlights a strong correlation between gut dysbiosis and IBD, antibiotics are now being employed to reestablish a balanced gut microbiome environment 2. However, existing drug therapies fall short of fully curing IBD, and prolonged usage often results in significant adverse reactions 3,4. In addition, while fecal microbiota transplantation is a burgeoning therapeutic technique, our grasp of its potential remains nascent. The evolution of individualized intestinal microbiota may offer a more hopeful avenue in the future 5. Recent studies on the influence of intestinal microorganisms on the dynamic balance mechanism of intestinal mucosa is gradually increasing. The dysbiosis of intestinal microbiota is closely associated with the normal function of the superficial barrier of the colon and the occurrence and development of IBD. Therefore, the potential of probiotics to modulate gut microbiota, bolster the immune system, and rejuvenate epithelium barrier function points towards novel avenues in the diagnosis and treatment of IBD 6.

**Figure 2:**
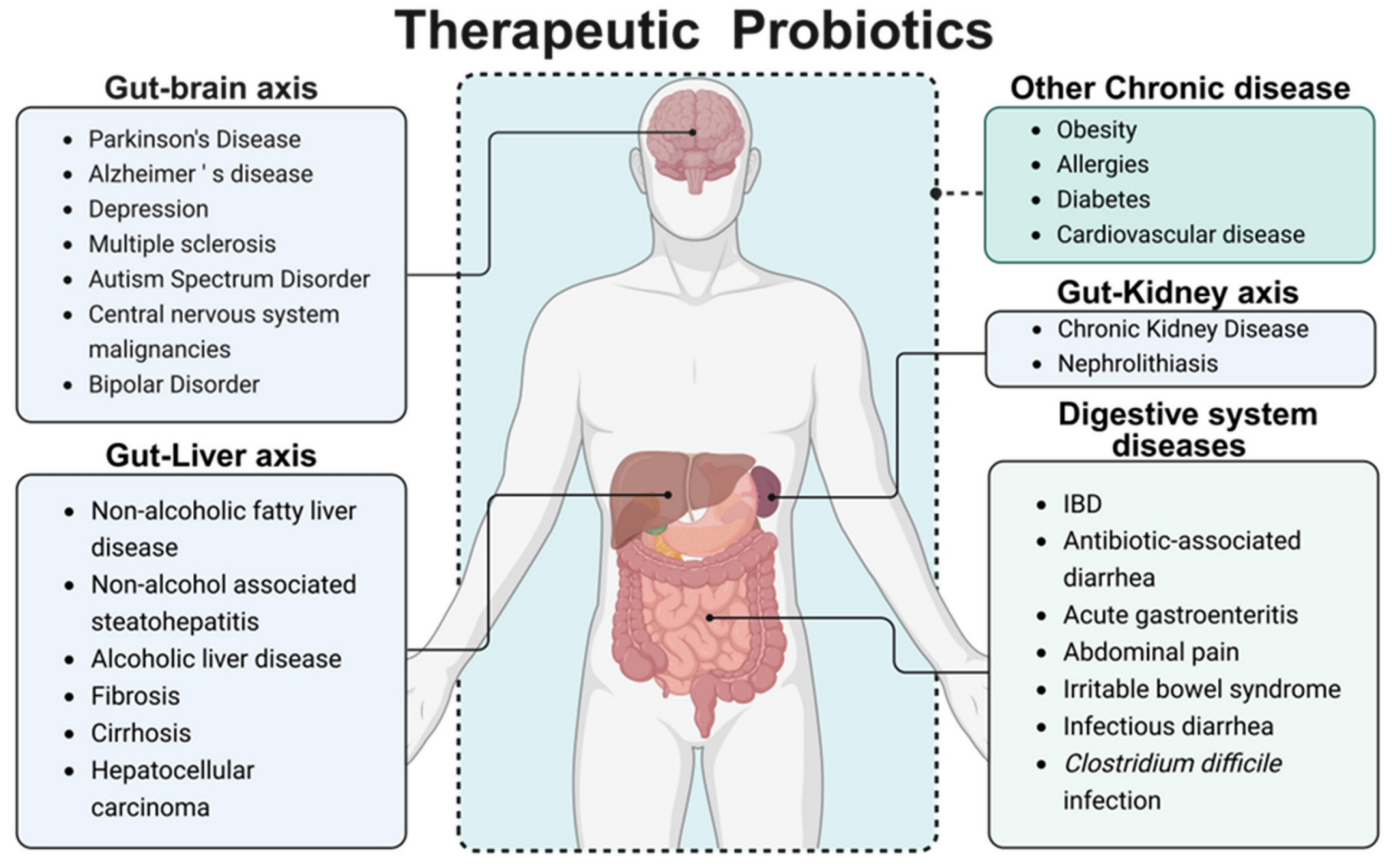
Therapeutic probiotics. Intestinal microbiota mainly regulates other organs and systems of the body through metabolic pathways (such as secretion of SCFAs, bacteriocins, etc.), immune pathways (stimulating the intestinal tract to maintain normal immune homeostasis and regulate immune factor levels), and neural pathways (affecting neurotransmitter levels). Multiple pathways are mixed with each other. Dietary intervention or exogenous supplementation of probiotics, prebiotics, or synbiotics can help to achieve adjuvant therapeutic effects on various diseases. Created with https://BioRender.com.

Probiotics are defined as live microorganisms that are beneficial to the human body when consumed in sufficient amounts 7. Probiotics mainly include two categories, *Lactobacillus* and *Bifidobacterium*. In addition, based on their benefits to the human body, some Gram-positive bacteria (such as *Lactococcus lactis*, *Streptococcus thermophilus*, *Enterococcus faecalis* and *Bacillus licheniformis*) and yeasts (such as* Saccharomyces boulardii*) are also classified as probiotics. The benefits provided by different probiotic strains vary significantly, with distinct mechanisms underlying each strain's action. Given the variability in gut microbiota composition between individuals, the efficacy of a particular probiotic strain can differ substantially. Current research suggests that probiotics residing in the human gut exert critical regulatory effects on immune function, the gut microbiota environment, intestinal barrier integrity, and even the function of other organs. These effects are mediated through probiotic-host interactions and the secretion of enzymes, organic acids, and small bioactive molecules 8. It has been widely used in the treatment of abdominal pain, diarrhea, hypertension, diabetes, and other diseases. Given the unique intestinal colonization characteristics of certain bacteria, utilizing specific probiotic strains as biological drug delivery carriers can partially circumvent the limitations associated with traditional drug delivery methodologies in IBD treatment, such as systemic adverse effects and poor drug targeting 9, thereby demonstrating substantial therapeutic potential for the healing of intestinal disease 10. Subsequent research has illuminated probiotics' capacity to modulate immune responses, maintain intestinal flora equilibrium, and yield pronounced effects in treating acute gastroenteritis 11, IBD 12, antibiotic-associated diarrhea 13, and diarrhea in children with abdominal pain 14, as well as irritable bowel syndrome 15 and *Clostridium difficile* infection 16. Moreover, probiotics' therapeutic potential extends to some central nervous system diseases, liver and kidney diseases through the gut-organ axis pathway 17-19; meanwhile, probiotic have certain therapeutic effects for other common chronic disease like obesity 20, allergies 21, diabetes 22, and cardiovascular disease (**Figure 2**) 23.

In this review, we delve into the cellular-level pathological alterations of IBD and discuss factors that could influence the disease. Our primary focus lies on probiotics, exploring their therapeutic mechanism in IBD treatment. The intricate interplay between the host organism and probiotic is discussed, with a focus on immunomodulation, antioxidants, intestinal barrier repair, and microbial environmental regulation. Further, we discuss the prevailing targeting strategies and standard methods for the multifunctional transformation of probiotic-based materials. Additionally, we present an in-depth analysis of two well-researched commercial probiotic formulations: VSL#3^®^ and LGG^®^. The review culminates with an exposition of recent applications of probiotics and prospective trajectories for probiotics within the domain of IBD treatment and beyond, casting a scientific lens on future directions and potential innovations.

## 2. Treatment mechanism

**Figure 3:**
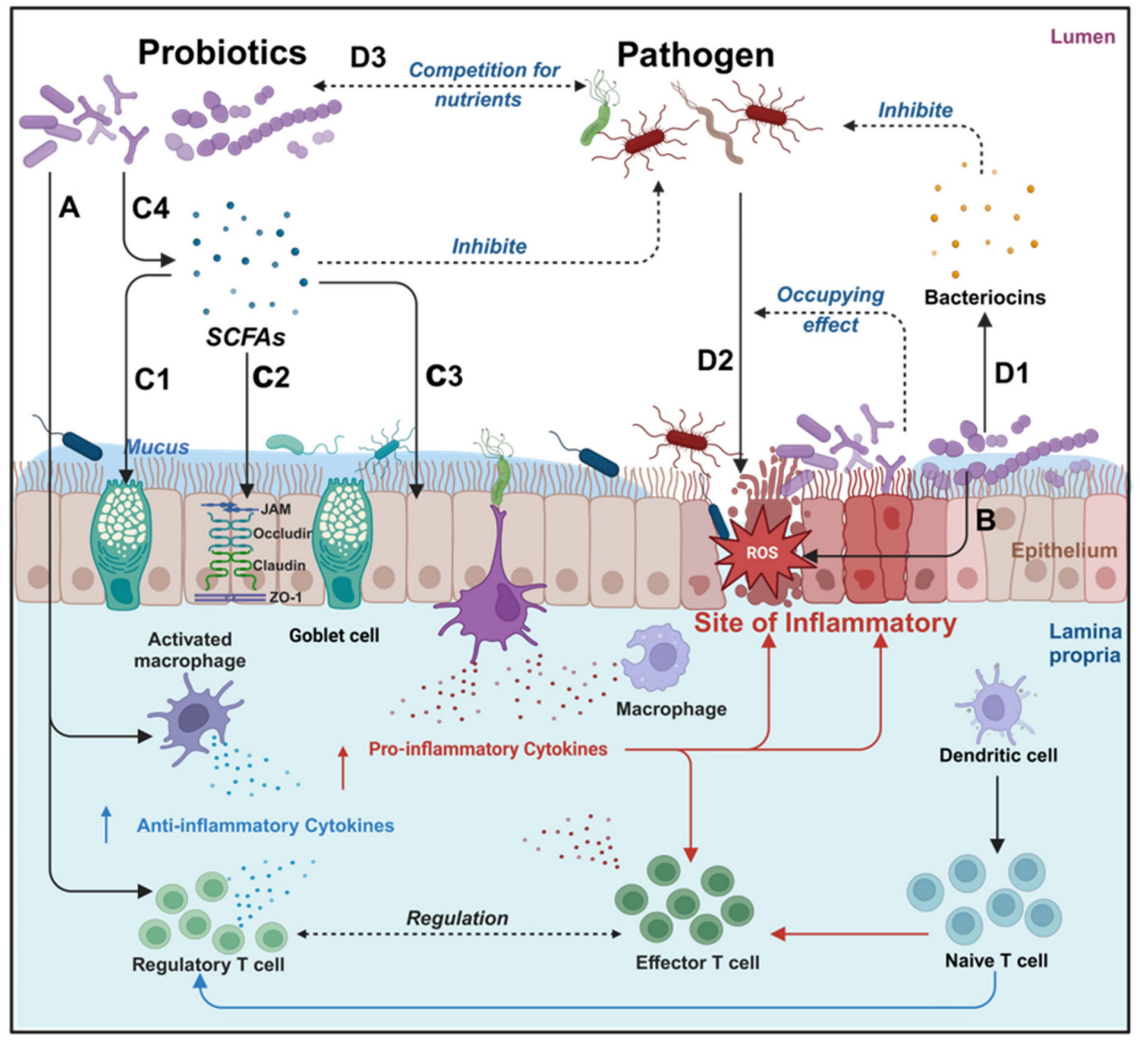
Overview of the main mechanism of probiotics in the treatment of IBD. Probiotics have various therapeutic effects on IBD, different probiotics may have different degrees of therapeutic effects, it can be roughly divided into immune regulation, antioxidant, anti-inflammatory, repairing intestinal barriers, and helping the body resist pathogenic microorganisms. **A:** Immunomodulation: promotion of anti-inflammatory factor expression and inhibition of pro-inflammatory factor expression; **B:** Neutralization of ROS; **C:** Probiotics can upregulate mucus protein secretion by goblet cells (**C1**), enhance tight junction protein function (**C2**), and intestinal epithelial cell function (**C3**) by secreting SCFAs (**C4**); **D:** Probiotics can inhibit pathogenic bacterium growth by the Secretion of bacteriocins (**D1**) and reduce pathogenic bacteria adhesion through occupancy effect (**D2**). Created with https://BioRender.com.

Probiotics manifest a spectrum of health benefits, contingent upon specific strains and the prevailing conditions within the gut microbiota. Probiotics can affect the human body through various mechanisms, including enhancement of digestion, nutrient secretion, modulation of neurotransmitter release, attenuation of pathogenic bacteria virulence, reduction in intestinal adhesion of pathogenic bacteria, synthesis of anti-inflammatory factors, neutralization of pro-inflammatory factors, facilitation of intestinal barrier restoration and modulation of intestinal flora 24. As shown in **Figure 3**, probiotics have demonstrated substantial therapeutic potentials in mitigating intestinal inflammation, exhibiting antioxidants, promoting intestinal barrier restoration, and regulating intestinal flora (**Table 1**).

### 2.1. Immunoregulation

Contemporary research posits a connection between both innate immune deficiencies and aberrations in adaptive immunity with IBD, albeit with the specifics remaining elusive. A plethora of immune alterations in IBD patients defy clear categorization as causes or effects of the disease 25,26. An upregulation of toll-like receptor 2 (TLR2) and TLR4 expression has been documented in active IBD cases 27, correlating with intestinal inflammatory infiltrates associated with helper T cells (Th cells), and regulatory T cells (Treg cells) 28. Furthermore, an elevation in antimicrobial antibodies in IBD patients indicates the stimulating effect of gut microbiota on the immune system and its associated pathologies 29. Inflammatory mediators hold a pivotal role in the progression of IBD.

Several studies conducted on probiotics have demonstrated their capacity to modulate TLR signaling and Th cell differentiation to Th1, Th2, and Th17 effector cells. This modulation results in a downregulation of pro-inflammatory factors 30, concurrently uplifting the levels of anti-inflammatory factors, thereby ameliorating symptoms of intestinal inflammation 31. The current research results indicate that probiotics can exert various regulatory effects on the immune system, mainly by regulating immune cells, inflammatory pathways, and immune mediators. Depending on the difference of strain, probiotic strains 32-35, surface layer proteins (Slp) 36-39, secretions 40, and extracellular vesicles 41,42 have all shown instances of immune regulation. Moreover, probiotics have been developed as efficient platforms for genetic engineering 43. Recombinant strains have been engineered to synthesize and secrete a range of anti-inflammatory factors, as well as neutralizing antibodies targeting pro-inflammatory factors 30,44-46, highlighting their significant potential in the treatment of IBD. Dou *et al*. demonstrated that oral administration of the *Lactobacillus* casei ATCC 393 strain and its metabolites significantly alleviated dextran sulfate sodium (DSS)-induced UC symptoms in mice, concurrently reducing inflammatory cytokine levels and immune cell infiltration 47. Hao *et al.* revealed that the anti-inflammatory effect of Lactobacillus plantarum Q7 is achieved through its secreted extracellular vesicles. After oral administration of extracellular vesicles, the pro-inflammatory factors in the serum were significantly reduced 42. Chandhni *et al.* extracted surface proteins from *Lactobacillus plantarum* MTCC 5690, *Lactobacillus fermentum* MTCC 568, and *Lactobacillus acidophilus* NCFM, revealing their respective degrees of anti-inflammatory effects 37. Luerche *et al.* emphasized the ability of *Lactococcus lactis* NCDO 2118 to increase the number of Treg cells and anti-inflammatory dendritic cells (DCs), inhibit interleukin-1β (IL-1β)-induced secretion of interleukin-8 (IL-8) by caco-2 cells, and demonstrate its anti-inflammatory activity *in vitro* cultured intestinal epithelial cells and DSS-induced UC mice models 48.

### 2.2. Antioxidant

**Figure 4:**
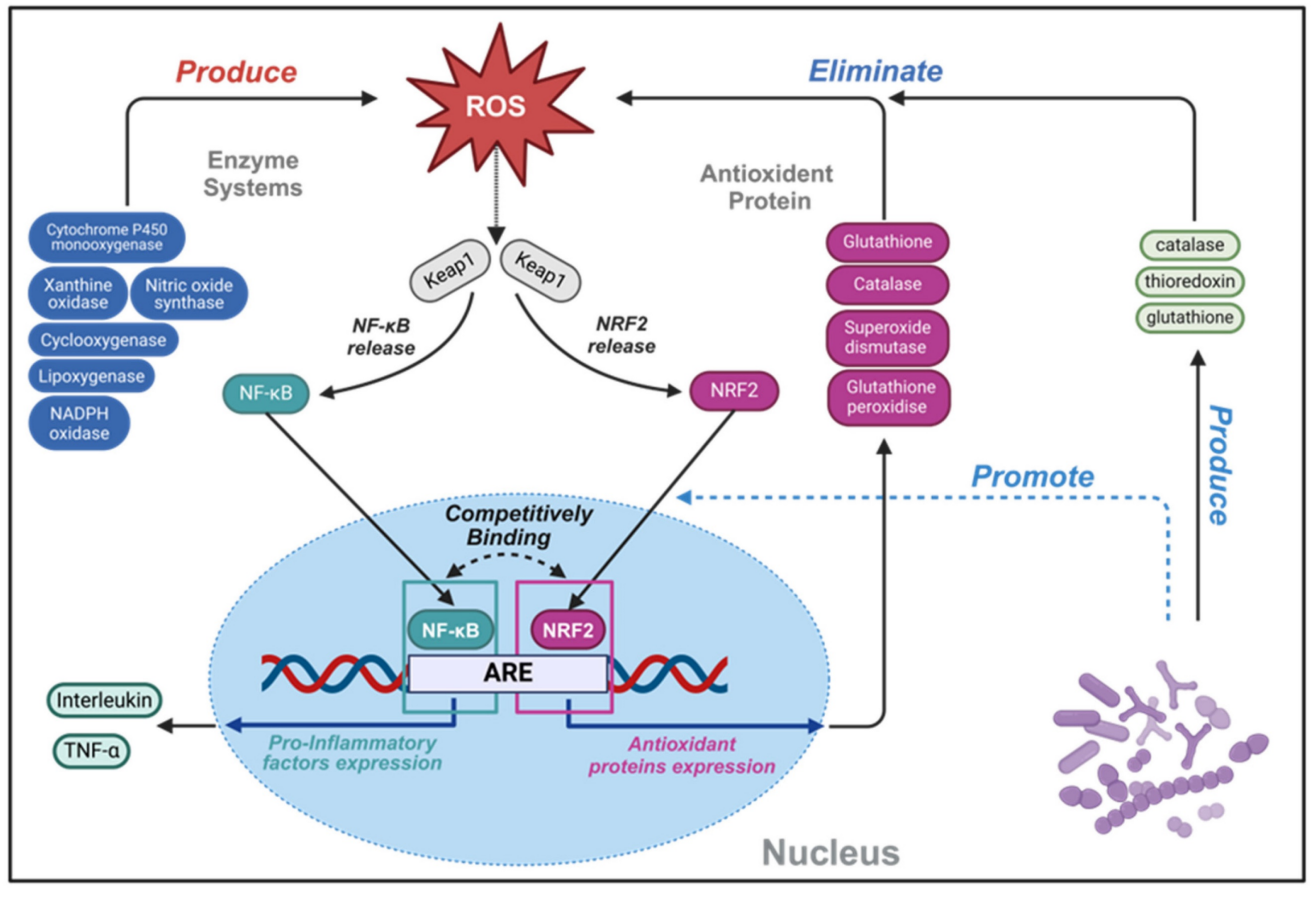
Schematic illustration of complex oxidant interaction mechanism between human cell and probiotics, highlighting the intricate interplay involving ROS. An increase in ROS can directly oxidize Keap1 protein, resulting in the escape of NRF2 and NF-κB from the inhibition of Keap1, entering the nucleus, and initiating an antioxidant stress response. Within the nuclear domain, NRF2 binds to ARE, which can start the transcription of a series of antioxidant enzymes and detoxification enzymes. This transcriptional activity culminates in a reduced production of ROS and mitigation of oxidative damage, thus inhibiting activation of NF-κB and production of inflammatory factors. Conversely, NF-κB can enters the nucleus and binds to the NRF2 promoter region, inhibiting the transcription of NRF2. Upon interaction with ARE, NF-κB will activate the transcription of inflammatory factors and immune related genes, thereby triggering inflammation and immune responses. Secretions from specific strain promote NRF2 pathway expression, while their secreted antioxidant enzymes synergistically enhance cellular antioxidant capacity. Created with https://BioRender.com.

Reactive oxygen species (ROS), predominantly arising as metabolic byproducts from mitochondria, peroxisomes and xanthine oxidase, consisting of radicals and nonradical derivatives of oxygen 49. Although ROS are crucial at moderate levels for maintaining immune function, defending against infection, and synthesizing thyroid hormone, a delicate balance is required to sustain normal cellular functions. Disruptions in ROS homeostasis may cause cellular injury, potentially culminating in disease and caducity 50. In the intestinal environment, an optimal concentration of ROS is pivotal for stimulating the proliferation and renewal of intestinal epithelial cells, maintain normal intestinal barrier function. Phagocytes, mediated by formyl peptide receptors (FPRs), internally produce high levels of ROS, crucial for the eradication of pathogenic microorganisms 51. ROS have been implicated in the etiology of inflammation, numbers of factors including intestinal epithelial cells alteration, genetic susceptibility, unregulated mucosal immune system, intestinal microflora dysbiosis, environmental factors, contribute to the development of IBD by influencing the levels of ROS. Furthermore, prolonged oxidative stress exposure can lead to sustained high-level DNA damage, which probably plays a key role in the pathogenesis of IBD 52. During the inflammatory progress, contact with gut immunogen stimulates intestinal epithelial cells, neutrophils and macrophages can produce ROS, inflicting damage on the intestine barrier. This disruption facilitates further antigen interactions with gut-associated lymphoid tissue and resident immune cells, escalating the secretion of pro-inflammatory cytokines and recruiting additional immune cells. Without timely intestinal barrier restoration, escalating ROS levels perpetuate cellular damage, fueling a vicious inflammatory cycle. Typically, the antioxidant mechanism of probiotic can be broadly categorized into two classes: the transcriptional upregulation of antioxidant proteins and the modulation of inflammatory signaling pathways. These multifaceted strategies underscore the therapeutic potential of probiotics in mitigating oxidative stress and interrupting the inflammatory cascade characteristic of IBD (**Figure 4**).

#### 2.2.1. Transcription and expression of antioxidant proteins

The antioxidant defense mechanism within probiotics encompasses three predominant enzyme-related antioxidant systems: catalase (CAT), thioredoxin-dependent and glutathione-dependent antioxidant systems 53. Depend on their specific living environment, various strains have evolved many antioxidant proteins to combat oxidative stress. These mainly include thioredoxin, thioredoxin reductase, CAT, heme-dependent CAT 54, which provide extra competitive advantage compared to other bacteria. In addition to the intrinsic antioxidant capabilities of bacteria, they are known to promote the host expression level of antioxidant protein-related genes 55. On the other hand, the introduction of exogenous genes coding for antioxidant enzymes into strains can also enhance the antioxidant capacity of bacteria. Carmen *et al.* demonstrated that oral administration of recombinant superoxide dismutase (SOD) and CAT-producing *Streptococcus thermophilus* CRL 807 exhibit enhanced anti-inflammatory activities on trinitrobenzene sulfonic acid (TNBS)-induced colonic tissue injury mouse model 56. Watterlot *et al.* revealed that intragastric administration of a SOD-producing recombinant *Lactobacillus casei* BL23 performed better anti-inflammation effects compared to both the standard BL23 strain and SOD treatment alone in DSS-induced colitis mouse model 57. Moreover, research conducted by Pan *et al.* indicated that *Lactobacillus plantarum* ZS62 was capable of upregulating the expression levels of SOD and CAT, thereby preventing and alleviating the symptoms of DSS-induced IBD in mouse model 58.

#### 2.2.2. NRF2 and NF-κB signal pathway

Probiotics employ additional antioxidant mechanisms through modulating the ROS signaling pathways nuclear factor erythroid 2-related factor 2 (NRF2) and nuclear factor kappa-B (NF-κB), both of which play critical roles in the emergence, development, and resolution of ROS-induced inflammation, as depicted in **Figure 4** (which delineates the interplay between ROS, NRF2, and NF-κB signaling pathways) 59. In the nucleus, NRF2 binds to antioxidant response element (ARE) sequences, thereby promoting the transcription of genes, including those encoding the antioxidative enzymes previously described. The NRF2 pathway encompasses a comprehensive set of genes related to ROS detoxification and pro-restitutive function and is recognized as the major regulatory mechanism of organisms to resist environmental oxidative stresses 59,60. Notably, NRF2-deficient mice exhibit heightened sensitivity to DSS, illustrating the pathway's protective role in colitis models 61.

Conversely, NF-κB represents a pivotal mediator in regulating immune and inflammatory processes, with its activation by ROS leading to the production of pro-inflammatory factors. The abnormal regulation of NF-κB can result in immune dysfunction and inflammatory disease 62. NF-κB played a protective role in maintaining the integrity of the intestinal barrier and immune homeostasis in IBD patients 63. The complex relationship of the crosstalk NRF2 and NF-κB signal pathway is highlighted by studies reporting both synergistic and antagonistic interactions between them 64. Furumoto *et al.* identified that 10-Oxo-trans-11-octadecenoic acid produced by *Lactobacillus plantarum* AKU1009a was able to significantly increase the level of NRF2 protein in HepG2 cells and promote the gene expression of a series of related antioxidant enzymes, a phenomenon also observed in mouse organs treated with oral administration of this substance 40. Another strain, *Lactobacillus plantarum* FC225, isolated from fermented cabbages, stimulated antioxidative enzyme gene expression in high-fat diet mice via NRF2-dependent transcriptional activation of ARE sites, enhancing the enzyme activities of SOD and glutathione peroxidase (GSH-px) 65.

The Slp of *Lactobacillus acidophilus* NCFM has been shown to inhibit lipopolysaccharide-induced inflammation through mitogen-activated protein kinase (MAPK) and NF-κB signaling pathways in RAW264.7 cells 36. Additionally, *Lactobacillus casei* Shirota has demonstrated the ability to adhere to differentiated intestinal cell-like caco-2 cells, reducing cell damage induced by oxidation of 2,2'-azobis (2-aminopropane) dihydrochloride and inflammatory stress by inhibiting the NF-κB inflammatory pathway 55.

### 2.3. Intestinal barrier repair

The intestinal barrier of the gastrointestinal tract demarcates human body from external environment, establishing a crucial platform for normal substance exchange between the body and the outside world. This barrier is comprised of mucus, epithelial cells, lamina propria. The development of IBD is often accompanied by a continuous impairment of intestinal barrier function and an increase in intestinal permeability 66. Pathogens can secrete enzymes to decompose tight junction (TJ) proteins between cells to increase gut barrier permeability 67. Furthermore, certain inflammatory cytokines also disrupt TJs, undermining the integrity of the gut barrier and facilitating the deeper tissue invasion by pathogenic bacteria.

#### 2.3.1. Mucus

Occupying the outermost layer of the intestinal barrier, mucus is primarily made up of water (90%-95%), electrolytes, lipids (1%-2%), proteins and others 68 formed by the substance secreted from epithelial cells, mainly goblet cells. It is constituted by 21 distinct types of mucins (MUC), denoted as MUC 1 to MUC 21 69. The core component of mucus is MUC 2, secreted by goblet cells, acting as a biological lubricant between epithelial cells and luminal microorganisms. It mitigates the direct interaction of gut cells with toxic substances and pathogens, thereby safeguarding them from infection and the impact of digestive juices. While gut microbiota can degenerate MUC proteins in mucus for their physiological activities, adhere and colonize to the epithelial surface, probiotics can increase mucus thickness and occupy the mucus binding sites. On the contrary, pathogens decompose mucin extensively, diminishing mucus thickness. It is noteworthy that various factors, including short chain fatty acids (SCFAs), bacterial components (flagellin, lipoteichoic acid, lipopolysaccharides), can influence MUC expression, stimulating goblet cells to secrete more mucus 68.

It is reported that Goblet cell depletion was observed in patient with IBD frequently, combined with mucus layer deficiency 70. Due to the amounts of MUC-degrading bacteria increase, the secretions of MUC 2 decrease and the levels of MUC 2 O-glycans change 71. These perturbations allow more microbiota to reach enterocytes, provoking immune responses, further increasing intestinal barrier permeability and exacerbating inflammation 72. *Akkermansia*, a current research hotspot, has shown a unique impact on mucus production compared to other probiotics. Although classified as a mucin-degrading bacterium capable of breaking down proteins, studies indicate that the Amuc_1100 protein expressed on its surface can stimulate goblet cell proliferation, enhance mucin secretion, and reinforce the barrier function of the intestinal epithelium 38.

#### 2.3.2. Epithelial cells and tight junction protein

Situated beneath the mucus layer, intestinal epithelial cells create a boundary between the external environment and the lamina propria. Enterocytes, hyperpolarized epithelial cells constituting the majority of the epithelial cell population, are interconnected by TJ proteins, crucial for maintaining the structural integrity of the intestine. These proteins include occludin, claudins, zonula occludens, tricellulin, cingulin, and junctional adhesion molecules, play an indispensable role in maintaining the integrity of the intestinal barrier and maintaining a reasonable direction of intestinal permeability disruptions in TJ protein expression and apoptosis of intestinal epithelial cells are commonplace in IBD, often culminating in altered intestinal permeability 60,73,74. Current research has shown that probiotics can upregulate the expression of TJ protein in various ways to protect epithelial cells, and reduce intestinal permeability. Al Sadi *et al.* found that *Bifidobacterium bifidum* enhances the TJ barrier function of the mouse gut by activating the P38 kinase pathway and TLR2 dependent pathway 75. Another strain, *Bifidobacterium pseudocatenulatum*, protects the intestinal barrier by inhibiting the TLR4/NF-κB pathway, slowing down intestinal inflammation symptoms, and upregulating TJ proteins and mucus expression levels 76. Hsieh *et al.* found that the SCFAs metabolites of the *Bifidobacterium* W1U2 strain can reduce the damage caused by tumor necrosis factor-α (TNF-α) to intestinal epithelial cells and help maintain the integrity of epithelial cells 77. In another study, *Lactobacillus gasseri* ATCC33323 regulated the intestinal barrier through constitutive androstane receptor-NR1I3, maintaining the localization of the E-cadherin/β-catenin complex and the E-cadherin/p120 catenin complex, reducing intestinal wall permeability, when E-cadherin was semiknocked out in the mouse intestine, the regulatory ability of *Lactobacillus gasseri* ATCC33323 was significantly reduced 78. Mazen *et al.* collected 11 *Lactobacillus* and *bifidobacteria* strains, discovering that 5 strains significantly ameliorated intestinal barrier function and restored TJ protein levels to varying degrees in TNBS-induced mouse model 79. The presence of micro-integrins on the cell wall surface of *Lactobacillus plantarum* CGMCC 1258 could repair TJ damage by increasing the expression of TJ proteins such as JAM-1, occludin and claudin-1 80.

#### 2.3.3. SCFAs

Fatty acids can be classified by the numbers of carbons in the main chain, as short-chain (<6 carbons), medium-chain (6-12 carbons), long-chain (12-21 carbons), or very long chain (>22 carbons) fatty acids. Most SCFAs, including acetate (C2), propionate (C3) and butyrate (C4), in the human intestine arise from bacterial metabolism of dietary fibers, acting as prebiotics, with each prebiotic potentially influencing gut microbiota differently 81. The concentration of SCFAs at molar ratio in the human gut tract is approximately 60:20:20 for acetate: propionate: butyrate 82. It has been reported that SCFAs have an important influence on maintaining normal function of the gut barrier. For instance, acetate has demonstrated the ability to suppress TNF-α secretion in lipopolysaccharide-induced mouse-derived blood cells through the g protein-coupled receptor 43 (GPR43) pathway 83 and to activate *Lactobacillus* bacteriocin synthesis by controlling quorum sensing 84; butyrate can induce zonula occludens-1 (ZO-1) and occluding assemble through the adenosine monophosphate-activated protein kinase dependent pathway 85; also butyrate is the main energy source for the colon epithelial cells 86. Furthermore, SCFAs also associate with body immune system 87,88 and other organ out of gut through gut-organ axis pathway beyond the improvement of intestinal barrier function.

Several studies indicate fecal SCFAs levels reduce in active IBD patients 89,90, with higher levels observed in patients in remission periods compared to those in active phases of the disease 91. Geirnaert *et al.* collected six strains of butyrate-producing bacteria, supplemented *in vitro* to CD patient microbiota increased butyrate production and enhanced intestinal epithelial barrier integrity 92. Clinical experiment shows SCFAs enemas induce remission in specific subsets of UC patients 93, whereas histological improvement was observed in UC patients 94. However, current research on how SCFAs administration affects gut microbiome and immune system in IBD patients remains elusive, and more research needs to be done to explain the causal relationship between IBD and SCFAs.

**Figure 5:**
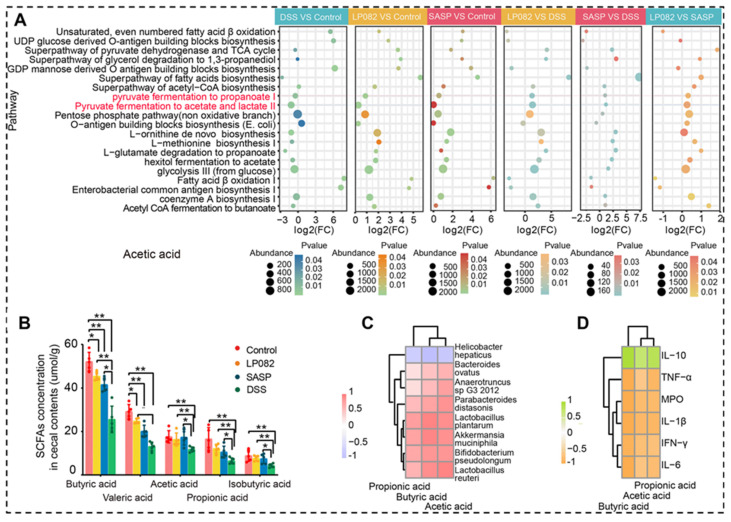
The important role of SCFAs in alleviation of DSS-induced UC. **A**: Gut microbial metabolic pathways associated with SCFAs. **B**: SCFAs contents. **C**: Relationship between SCFAs and gut microbiota. **D**: Relationship between SCFAs and inflammatory cytokines. LP082: *L. plantarum* HNU082 treated group; SASP: salazosulfasalazine treated group; DSS: colitis group. Reproduced with permission from 95, copyright 2022, ASM Journals.

As SCFAs-producing gut bacteria, *Lactobacillus* and *Bifidobacterium* generally produce lactic and acetic acids as primary end products of carbohydrate metabolism. Wu *et al.* revealed the intake of *Lactobacillus plantarum* HNU082 upregulated the level of SCFAs, which is strongly negatively correlated with the proinflammatory factors, but strongly positively correlated with the inflammatory suppressor, the relative abundance of SCFAs-producing bacteria is also increased (**Figure 5**) 95.

### 2.4. Microbial environmental regulation

Mounting evidence suggests a close association between gut microbiota and the progression of IBD 96. The pathogenesis of IBD cannot be attributed to a single intestinal microorganism; instead, it is characterized by a gradual decrease in the overall diversity of intestinal flora as the disease progresses 97. An increased abundance of *Escherichia coli*, *Vibrio desulfuricans*, *Clostridium perfringens*, and *Enterococcus faecalis* have been found to be associated with high activity of IBD 98. A key question that is still waiting for an answer is the causal relationship between dysbiosis and IBD. Additionally, it should be pointed out that other members of the gut microbiome except bacteria, for instance, fungi 99, bacteriophage 100 and archaea 101, also contribute to the disease, though further research is required to elucidate their specific roles. The main mechanism of probiotic antagonistic effect on pathogenic bacteria includes producing bacteriocins, organic acids, competition for nutrients, and occupying effect.

#### 2.4.1. Bacteriocins

Bacteriocins, as peptides or proteins synthesized by bacteria exhibiting the ability to inhibit the growth of specific bacteria strains, are generally classified into two groups: peptides that undergo significant post-translational modifications (class I) and unmodified peptides (class II) 102. Due to their inhibitory effects on pathogens and low oral toxicity to their host, bacteriocins have potential as therapeutic agents against various infectious diseases. Detailed mechanisms of action have been extensively reviewed elsewhere 102. Given the crucial role of gut microbiota in IBD progression, antibiotics have been widely used in clinical settings and have proven to be effective 2. However, bacterial resistance and adverse effects pose limitations to antibiotic use, making bacteriocins a promising alternative.

#### 2.4.2. Competing and occupying effect

The human digestive tract offers a vast surface area for bacterial colonization, the number of bacteria is about 10^7^ in the jejunum, 10^11^ in the ileum, and 10^14^ in the colon. Due to differences in pH value and oxygen concentration, the composition of bacteria in different parts of the digestive tract varies, while the colon is the main contributor to the total population of bacteria in the alimentary tract 103. To avoid mechanical clearance effects by digestive fluid and food, bacteria have evolved many strategies to adhere to the target cell in the gut. Thus, adhesion to host cells is a prerequisite for bacterial survival and proliferation within the host.

Pili, which widely exists on the surface of most Gram-negative bacteria and some Gram- positive bacteria, plays an important role in the adhesion process between bacteria and host cells. Composed of thousands of pili proteins, the tip of it confers varying degrees of adhesion ability to other cells 104. Some bacteria also possess surface factors that can recognize various classes of host surface cell molecules, including components of the extracellular matrix, transmembrane proteins, exhibiting adhesion characteristics. The mutual binding process between bacteria and host cells is the critical preliminary step for bacteria invasion 105.

Compared to out-cell adhesion, survival within cells means the bacteria can partially avoid the competition from other members of the microbiome and gain more nutrients. Consequently, numbers of different strategies have been evolved to invade host cells by pathogens. These include bacterial surface proteins binding to host cell molecules, leading to bacterial engulfment, exemplified by *Listeria monocytogenes* 106 and the activation of cell signaling pathways by bacteria, causing cytoskeletal rearrangement of the cell membrane and engulfment of nearby bacteria, as seen in *Salmonella* 107. By entering M cells and DCs, pathogens can penetrate the epithelium barrier to facilitate its dissemination in the host 108.

Probiotics can prevent the colonization of pathogenic microorganisms by attaching to the surface of intestinal epithelial cells, thereby physically blocking them through occupying effect 102 competition for nutrients. Abdi *et al.* isolated 323 strains of *Lactobacillus* from healthy human breast milk, with 71.8% of the strains showing strong adhesion to intestinal epithelial cells 109. Another strains *Lactobacillus plantarum* BMCM12 can secrete extracellular proteins to reduce the adhesion capacity of pathogenic bacteria significantly and protect the intestinal barrier 41.

## 3. Strategies for engineering probiotic-based materials

In recent years, the field of bacterial modification has witnessed significant advancements with the development of a plethora of physicochemical and biological methods. Modified bacteria can be increasingly utilized as drug transporters or expression carriers for therapeutic interventions. However, the challenging conditions within the gastrointestinal tract, characterized by a harsh pH environment and the presence of various digestive fluids such as pepsin, pancreatic enzymes, and bile acids, pose substantial obstacles 110. Although specific strains are able to resist bile by producing bile acid salt hydrolase 111, the activity of most of the bacteria will still be greatly affected 112. At this stage, the main objectives of the modification of bacteria can be summarized as follows: Enhancing the gastrointestinal stability of the bacteria and augmenting their activity 112,113; Improving the targeting efficacy of the bacteria to ensure colonize and proliferate at specific sites 114; Endowing bacteria with the capability to express specific genes, facilitating the production of the required substances 115; Enabling the bacteria to bind to specific drugs, using their targeting properties for the targeted release of drugs 116,117. It is crucial to acknowledge that relying solely on the modification of the bacterial shell without editing its genes may lead to the dilution of the shell of the modified bacteria as the bacteria continue to divide and multiply, affecting the subsequent therapeutic effect.

In this section, we will delve into colon targeting strategies and bacterial modification methods, categorizing them into physicochemical and biological methods based on the materials and technologies employed. The classification criterion hinges on whether the method endows the bacteria with the ability to autonomously produce substances related to the modification target.

### 3.1. Engineering approaches for probiotic materials in targeting IBD

The inflammatory sites of IBD patients present significant different in their inflamed tissue sites compared to healthy individuals. These alterations encompass a lower pH value of the inflamed site (2.3~5.5) 118, a higher ROS and inflammatory mediator levels, higher degree of inflammatory cell infiltration and permeability of the colonic epithelium 119, prolonged transit time, reduced level of TJ protein and mucus secretion 120. Additionally, there is a variation in the distribution of intestinal bacterial flora 121. Leveraging these differences in pH, transit time, specific inflammatory cells, microbial environment, and ROS levels can facilitate the design of targeted therapy strategies for IBD patients 122,123.

**Figure 6:**
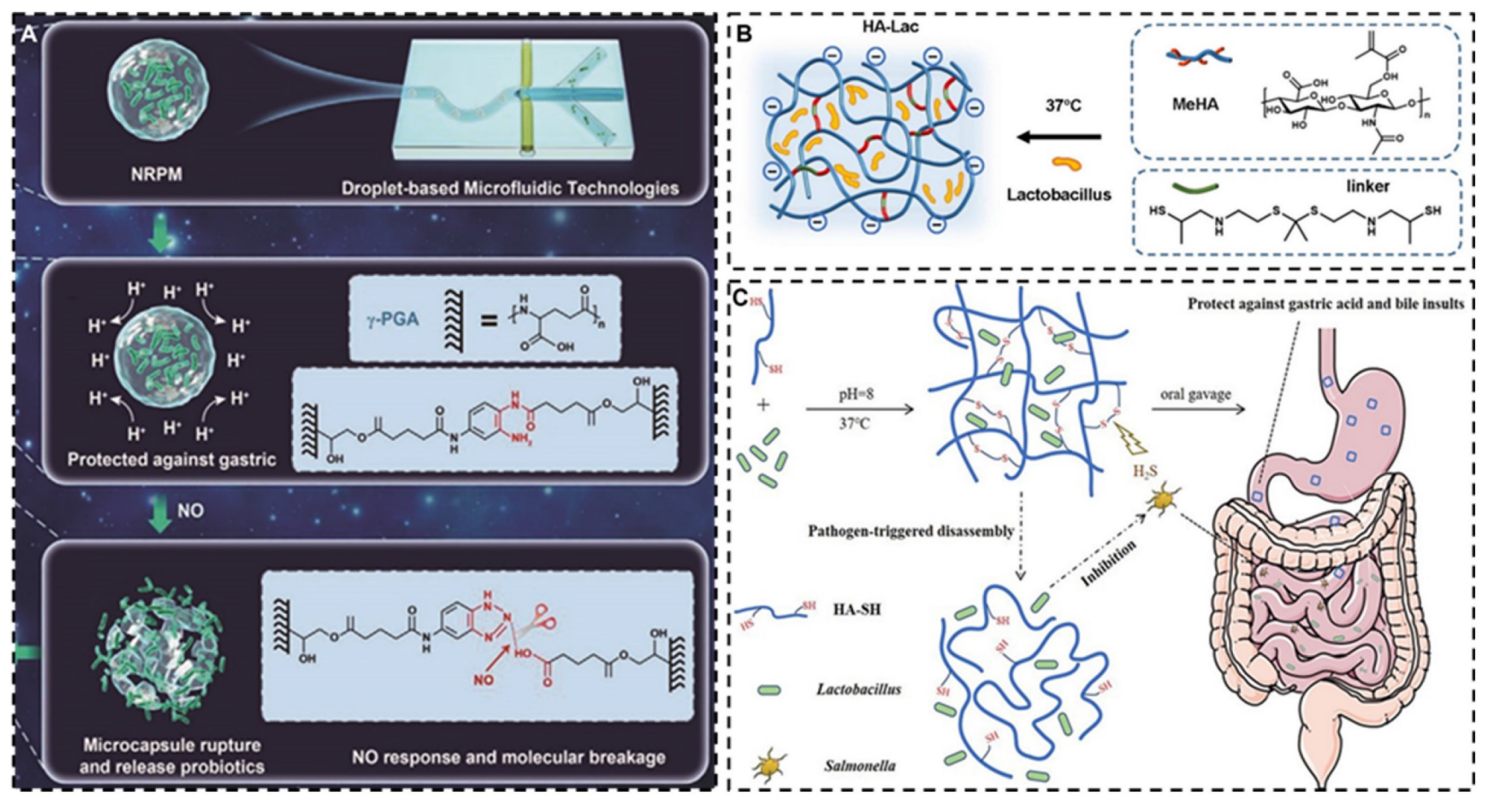
Schematic illustration of encapsulated probiotics based on different targeting strategies. **A:** NO-responsive poly-γ-glutamic acid hydrogel microcapsule. Reproduced with permission from 125, copyright 2022, John Wiley and Sons. **B**: ROS-responsive HA hydrogel. Reproduced with permission from 126, copyright 2022, Elsevier. **C**: H_2_S-triggered HA hydrogel. Reproduced with permission from 127, copyright 2020, America Chemical Society.

Although the intestinal transit time and pH environment of IBD patients are significantly different from those of the healthy population, considerable inter-patient variability exists. Furthermore, the diarrhea induced by intestinal inflammation can unpredictably alter drug transit times, challenging the achievement of desired therapeutic outcomes with traditional targeting methods. Consequently, it is comparatively more rational to devise targeting strategies based on the distinct characteristics of inflammatory sites, such as the ROS environment 124, inflammatory cell infiltration, and specific microbial milieu, which starkly differ from normal intestinal tissues. Wang *et al.* designed an NO-sensitive gather γ- glutamate microgel for encapsulating *Lactobacillus*, which endowed the modified strain with good stability to gastric acid and a good targeting effect on NO gas secreted by intestinal inflammatory tissues 125. Huang *et al.* developed a ROS-responsive hyaluronic acid (HA) hydrogel based on physiologically crosslinked methacrylated HA and thiolated thioketal, in which ROS selectively cleaved thioketal linkages to release *Lactobacillus reuteri* in the inflamed colon tissues 126. Xiao *et al.* use HA hydrogel wrap *Lactobacillus rhamnoses*, which can rapidly drop the drug locally upon contact with H_2_S produced by the metabolism of pathogenic bacteria, achieving a targeted therapeutic effect (**Figure 6**) 127. However, current research on bacterial modification predominantly focuses on non-targeted areas, with studies on targeted modification remaining scarce.

### 3.2. Physicochemical methods for engineering probiotic-based materials

The bacterial cell wall, directly interacting with the human immune system, has emerged as a focal point in bacterial modification research. Structurally, bacteria cell wall consists of two main components: the cell wall and the protoplasm. The cell wall mainly consists of a glycan skeleton (composed of N-acetylglucosamine and N-acetylcytidylic acid), tetrapeptide side chains and pentapeptide cross-linked bridges 128. The cell wall's surface, enriched with phosphopeptidic acid and peptidoglycan, is negatively charged and adorned with various functional groups. Through tailored physicochemical methods, chemical bonds can be formed between exogenous substances and cell wall functional groups, thus achieving surface modification that can achieve a variety of functions. The most prevalent mechanisms of bacterial physicochemical modification include electrostatic adsorption, wrapping, and the establishment of various covalent or non-covalent bonds.

**Figure 7:**
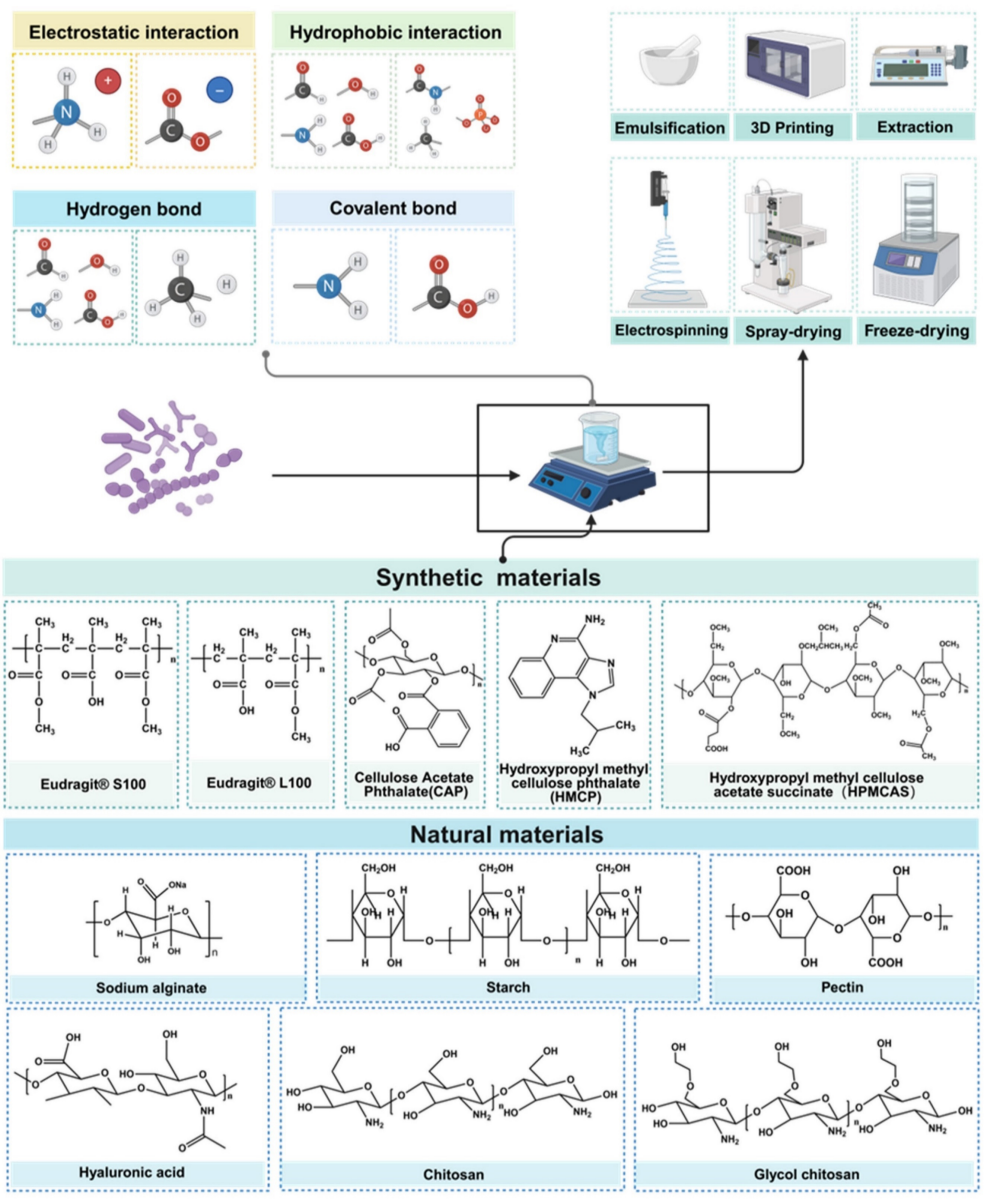
Illustration of molecular interaction, encapsulate methods and materials of engineered probiotics. Different encapsulation materials are combined with strains through various forces, and according to the different preparation methods, the strains exhibit various improvements in physical and chemical properties. Created with https://BioRender.com.

Beyond the modification of the bacterial strain itself, adept formulation and encapsulation technologies can enhance the digestive stability of the strain, mitigating the impacts of temperature, oxygen exposure, contact with encapsulation materials, and pressure during formulation, transportation and storage processes. Oral administration stands as the most common delivery route for probiotic products, which are conventionally formulated into tablets, granules, capsules, and other dosage forms. The main research directions include forming physical barriers to isolate the external environment, combining with prebiotics to improve strain viability, and combining with digestive enzyme hydrolases to improve strain digestive tract stability. The commonly used natural materials usually refer to natural polysaccharides and proteins, including soy protein 113, alginate 129, pectin 130-132, chitosan 133, whey protein 134, starch 135, silk sericin 136 and silk fibroin 137. Synthetic materials include the Eudragit^®^ series 138, hydroxypropyl methyl cellulose phthalate, cellulose acetate and other synthetic polymers (**Figure 7**) 139,140. Probiotic encapsulation technology can be categorized based on the relationship between the encapsulated bacteria and the encapsulation mechanism, falling into either block-based bulk encapsulation and single bacterial encapsulation.

#### 3.2.1. Bulk encapsulation

**Figure 8:**
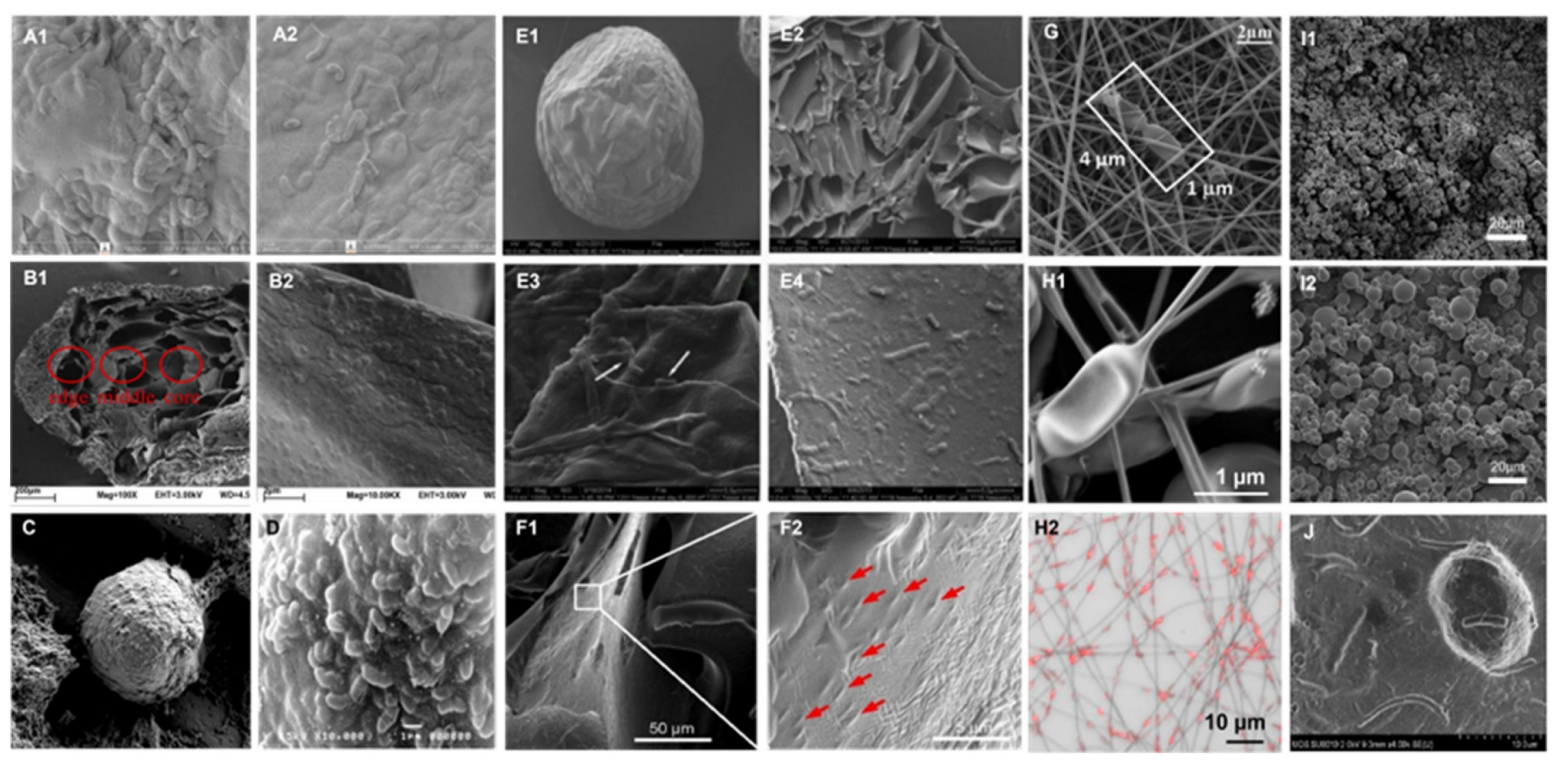
Images of bulk encapsulated probiotics with different materials and methods. SEM images of alginate-silk sericin-maltitol co-encapsulated *L. casei* TISTR 1463 with (**A1**) and without (**A2**) silk sericin coating. Reproduced with permission from 136, copyright 2022, Elsevier. SEM images of calcium pectin beads surface morphology (**B1**) and the distribution (**B2**) of *L. paraplantarum* L-ZS9 within it. Reproduced with permission from 132, copyright 2022, Elsevier. SEM image of encapsulated *L. rhamnosus* based on robocasting 3D-printing technology (**C**). Reproduced with permission from 144, copyright 2023, Elsevier. SEM image (**D**) of the surface of *L. casei* 01 beads coated with alginate plus hi-maize starch. Reproduced with permission from 135, copyright 2016, Elsevier. SEM images of hydrogel beads encapsulated with *L. rhamnosus* GG (LGG^®^) after freeze-dry: surface view (**E1, E3**) and fractural section (**E2, E4**). Reproduced with permission from 130, copyright 2023, Elsevier. SEM images of HA hydrogel-encapsulated *L. reuteri* (**F1, F2**). Reproduced with permission from 126, copyright 2022, Elsevier. SEM images (**G**) of electrospun LGG incorporated in fibrous mats from calcium caseinate-pullulan-LGG fibers. Reproduced with permission from 142, copyright 2022, Elsevier. SEM images (**H1**) and confocal microscopy images (**H2**) of *L. plantarum*-loaded poly(ethylene oxide) nanofibers. Reproduced with permission from 143, copyright 2019, Elsevier. SEM images of calcium-alginate (**I1**) and calcium-alginate-sucrose (**I2**) encapsulated LGG**^®^**. Reproduced with permission from 141, copyright 2022, Elsevier. UHR FE-SEM images of pectin resistant starch-pectic oligosaccharide hydrogel beads encapsulated *L. bulgaricus* (**J**). Reproduced with permission from 131, copyright 2023, Elsevier.

The bulk encapsulation is characterized by the embedding of a substantial number of bacteria within a protective matrix or their adherence to the substrate's surface, as discernible via electron microscopy. At the microstructural level, it can be divided into two forms, nanospheres and nanofibers. As shown in **Figure 7**, predominantly, the encapsulation techniques encompass include spray-drying 113,141, extraction 129,134, freeze-drying 130, emulsification 135, electrospinning 142,143, 3D printing 144. The chosen encapsulation method and substrate crucially influence the final encapsulation outcome. **Figure 8** exhibits images of bulk encapsulated probiotics with different materials and methods. Within nanospheres, bacteria and matrix are uniformly integrated, though chemical bond forces are not conspicuously dominant. The main advantages of bulk bacterial encapsulation technology are its capacity for encapsulating a large bacterial quantity, low production cost, expedited experimentation, and the simultaneous analysis of multiple bacterial responses, its production process is easier to control. Bulk encapsule technology is usually aimed at improving the shelf life, digestive stability, and storage activity of probiotics, rendering it a promising avenue for industrial application.

#### 3.2.2. Single bacterial encapsulation

**Figure 9:**
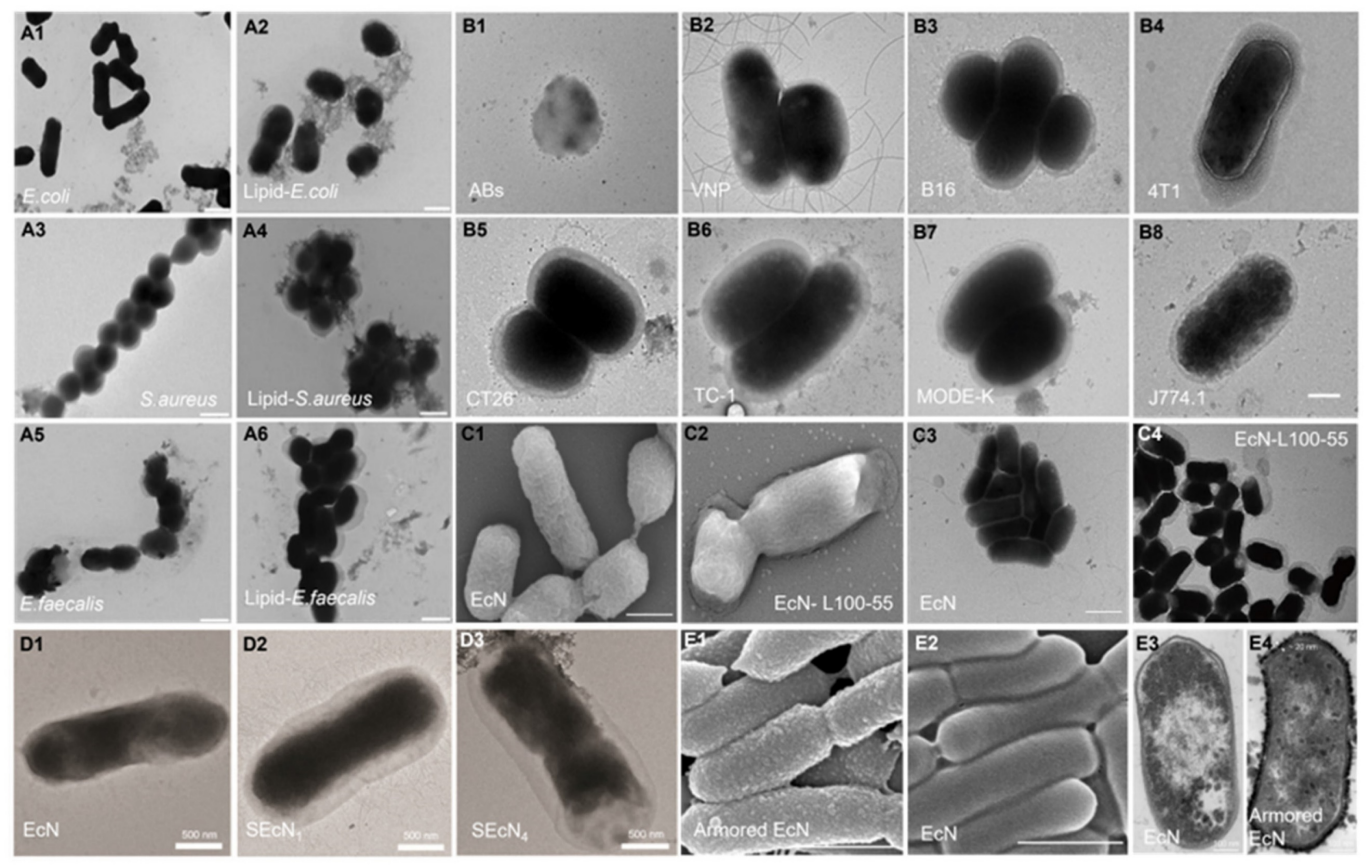
Images of single encapsulated bacteria with different materials and methods. TEM images of uncoated (**A1, A3, A5**) and Lipid coated bacteria (**A2, A4, A6**). Reproduced with permission from 151, copyright 2019, Springer Nature. TEM images of apoptotic bodies (**B1**, Abs), naked *Salmonella Typhimurium VNP 20009* (**B2**, VNP) and VNP coated with different tumor cell membranes (**B3-B8**). Reproduced with permission from 152, copyright 2022, Elsevier. SEM images of *Escherichia coli* Nissle 1917 (EcN) (**C1**) and EcN- Eudragit^®^ L100-55 (**C2**), TEM images of EcN (**C3**) and EcN-Eudragit^®^ L100-55 (**C4**). Reproduced with permission from 138, copyright 2020, John Wiley and Sons. TEM images of EcN (**D1**), ECN with 1 layer (**D2**, SEcN_1_) and 4 layers (**D3**, SEcN_4_) of silk fibroin. Reproduced with permission from 137, copyright 2021, John Wiley and Sons. SEM images of armored (**E1**) and naive EcN (**E2**), cross-sectional TEM images of naive (**E3**) or armored EcN (**E4**). Reproduced with permission from 146, copyright 2022, Springer Nature.

In contrast, single bacterial encapsulation typically involves surface coating of protective matrices on bacteria, with an association to the substrate via chemical bond forces or interaction forces, including covalent bonds, diverse electrical properties, hydrophobic interactions, Van der Waals forces, and intermolecular hydrogen bonding (**Figure 9**). This technology offers precision in bacterial encapsulation, fostering advancements in bacterial-based drug delivery, and providing the bacteria with complex functionalities such as drug delivery 145, multi-antibiotic resistance 146, transcending traditional roles in gastrointestinal tract protection and viability enhancement.

In this section, we broaden our exploration to specific single bacterial encapsulation technologies based on the solution self-assemble (SA) method categorized by different driven forces. Single bacterial encapsulation techniques utilize a variety of principles to construct molecular layered structures with differences primarily in encapsulation mechanisms and layer quantity. Characterized by its spontaneously ordered nanoparticle structuring in solution without external interference, SA relies on interactions such as electrostatic interaction 147,148, hydrogen bond 149, covalent bond 145,150. The SA method is a natural and spontaneous process that leads to the organized structuring of nanoparticles in a solution without the need for external intervention. Throughout this process, nanoparticles autonomously arrange themselves into ordered structures, facilitated by the interplay of hydrophobic forces, Van der Waals interactions, and electrostatic forces. The simplicity and ease of implementation of SA technology, along with its rapid and straightforward preparation process, result in structures that exhibit commendable stability. A distinct advantage of SA is its frequent application in decorating bacteria with biofilm and biomimetic materials, such as lipid 151, tumor cell membrane 152, which gives the bacteria many unique biological characteristics. However, the controllability and stability of SA technology require improvement. It necessitates the careful selection of appropriate conditions and molecules to regulate its assembly structure and properties.

Layer-by-Layer (LBL) method is one specific SA method driven primarily by Van der Waals interactions which provides researchers with the capability to precisely control the thickness of each layer, encapsulating bacteria with various materials and layer counts to create complex bacterial shells. During the LBL process, each layer requires chemical modification of the surface charge before deposition onto the substrate surface. The subsequent mutual adsorption between each layer through electrostatic attraction allows for precise control and adjustment. However, this also implies that the encapsulation procedure is more intricate and time-consuming, necessitating consideration of numerous control conditions, the mastery of advanced laboratory skills by researchers, and presenting challenges in large-scale production.

### 3.3. Chemical methods for engineering probiotic-based materials

**Figure 10:**
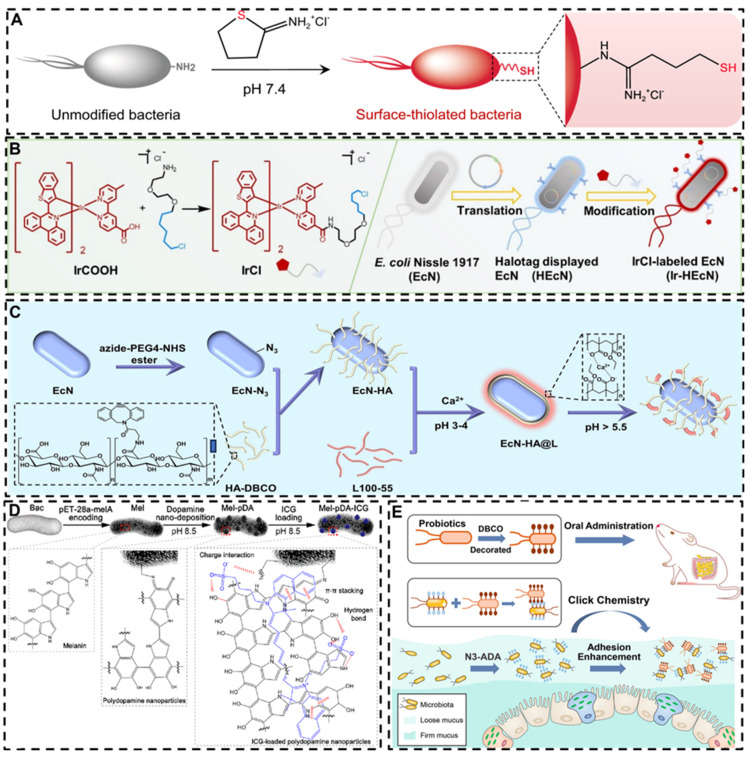
Schematic illustrations of chemically modified probiotics using different strategies and molecules. **A:** Surface-thiolated ECN. Reproduced with permission from 154, copyright 2022, Springer Nature. **B:** Iridium (III) photosensitizer-bacteria hybrid. Reproduced with permission from. Reproduced with permission from 155, copyright 2020, John Wiley and Sons. **C:** Bioorthogonal functionalized EcN. Reproduced with permission from 158, copyright 2024, Elsevier. **D:** Ternary photosensitive bacteria. Reproduced with permission from 156, copyright 2023, America Chemical Society. **E:** DBCO and Azido group modified bacteria. Reproduced with permission from 159, copyright 2022, America Chemical Society.

The bacterial cell wall is rich in functional groups, enabling the chemical conjugation of small molecules or other materials to the bacterial surface through a variety of bonding techniques. Such chemical modifications can be used to alter the surface properties of bacteria, similar to physical modification, which plays a crucial role in enhancing the ability of probiotics to target tumor and inflammatory sites, increasing bacterial resilience against external stressors, improving therapeutic efficacy, and augmenting intestinal colonization. The primary advantage of chemical modification lies in its ability to precisely introduce complex and diverse functional groups to the bacterial surface, thereby enabling a wider range of functionalities. Recent advances in click chemistry and bioorthogonal technologies have provided highly efficient and sophisticated tools for bacterial surface engineering (**Figure 10**). For example, Liu *et al.* attached PEG to the surface of bacteria by *in situ* self-polymerization of dopamine, amine-terminated polyethylene glycol (m-PEG-NH2) linked with self-polymerized polydopamine on the surface of bacteria significantly enhancing their ability to penetrate and retention at the intestinal mucus layer, as well as their mobility. Bacteria modified by this approach effectively inhibited pathogen invasion by occupying available niches 153. In another study from the same group, bacteria were surface-modified with thiol groups, allowing them to form covalent bonds with disulfide-rich mucins in the intestinal environment, leading to a remarkable 170-fold increase in mucosal adhesion 154.

In subsequent studies, researchers have combined chemical and biological techniques, employing plasmid expression to display specific molecules on the bacterial surface, followed by attachment of metal complexes and indocyanine green to enhance photothermal effects 155,156. Cao *et al.* synthesized SOD/CAT mimic antioxidant enzymes and combined them with the surface of bacteria through click chemistry reactions, a metal-organic-framework encapsuled iron single-atom SOD/CAT mimic catalyst was linked to the surface of bacteria through Click reaction with boronic acid-poly(ethylene glycol) (C18-PEG-B), providing the bacteria with additional antioxidant capacity, protecting them from inflammatory damage at the inflammatory site, and demonstrating significant therapeutic effects in mouse and beagle dog IBD models 157. Peng *et al.* used a click chemistry reaction of azide acetylene to bind HA to bacterial surfaces, the amino groups of the *Escherichia coli* Nissle 1917 cell wall were diazotization modification, and the diazo groups on the *Escherichia coli* Nissle 1917 surface are linked to dibenzocyclooctyne (DBCO) functionalized HA through a bioorthogonal reaction, then L100-55 were coated on the outer side, they demonstrated significant immunomodulatory and gut microbiota regulatory effects in mice model 158. Song *et al.* developed a modification strategy using bioorthogonal reactions, which modifies DBCO group onto the surface of probiotics and expresses azide groups on the surface of gut microbiota by using metabolic engineering methods. DBCO-functionalized probiotics can undergo bioorthogonal reactions with the gut microbiota, improve their intestinal colonization ability, enhance bacterial delivery efficiency. This technology may have enormous clinical translational potential 159.

### 3.4. Bioengineering methods for engineering probiotic-based materials

**Figure 11:**
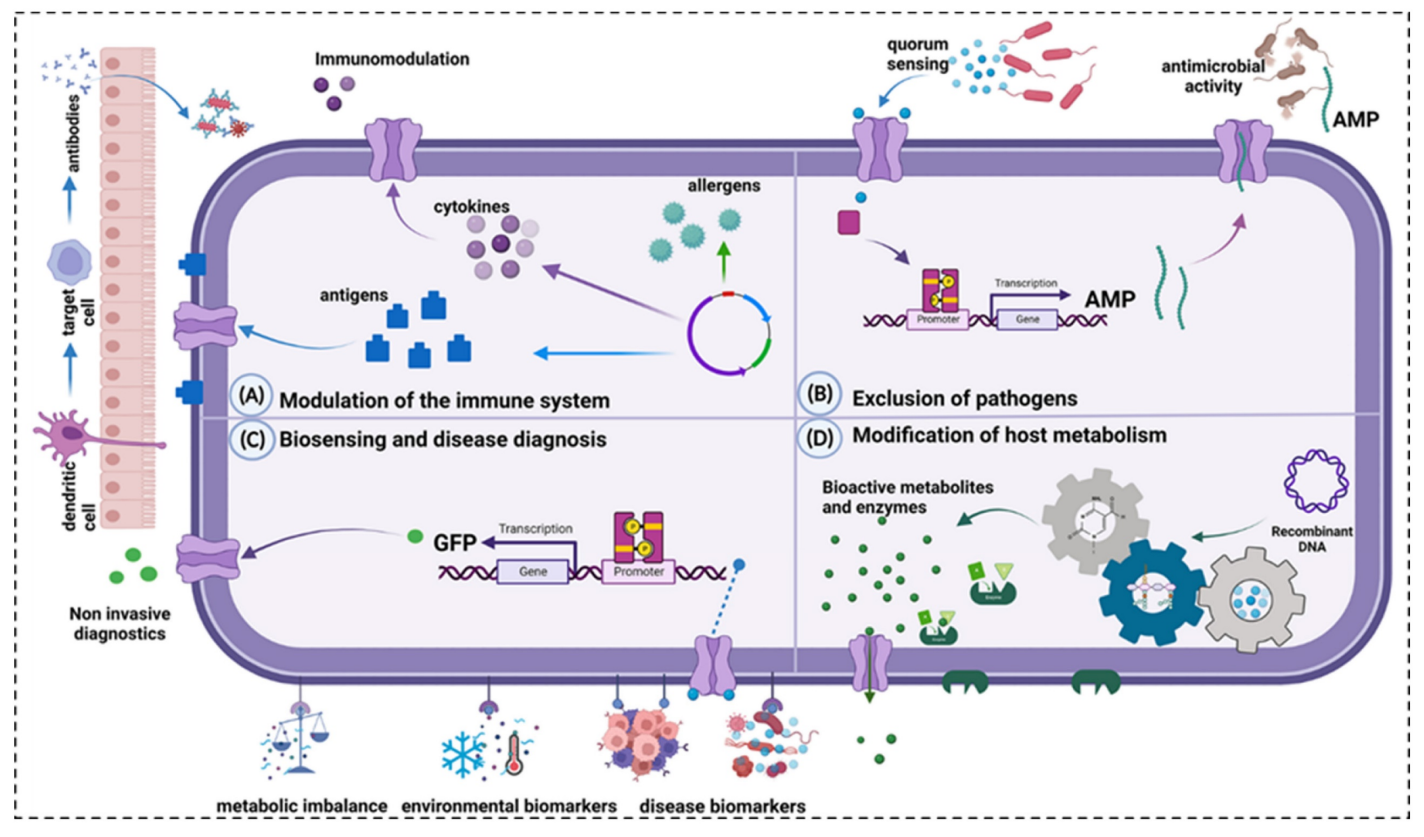
Main mechanisms of action of engineered probiotics. **A:** Modulation of the immune system. **B:** Exclusion of pathogens. **C:** Biosensing and disease diagnosis. **D:** Modification of host metabolism. Reproduced with permission from 161, copyright 2023, Portland Press, Ltd.

Bioengineering methodologies facilitate the genetic manipulation of probiotics empowering them to express, synthesize, and secrete various specific therapeutic substances *in situ*. This approach circumvents the loss and some adverse effects associated with the traditional oral administration of drugs through the digestive tract, as illustrated in **Figure 11**, which delineates the primary mechanisms of action of engineered probiotics in disease management. However, it is critical to acknowledge that many strains suitable for genetic modification are not endogenous to the human gut microbiota. The competitive survival dynamics within the human gastrointestinal ecosystem can impact the colonization efficiency of engineered probiotics, subsequently diminishing their therapeutic potential 160. Thus, selecting appropriate strains as platforms for genetic modifications becomes imperative 161.

Gene editing techniques have been widely used in the gene transformation of bacteria, for instance, using plasmids and phages as gene carriers to import exogenous genes 162. CRISPR/CAS gene editing technology has also been used to achieve this goal, which can express a variety of products 163,164. While plasmid-based methods offer simplicity and a broad selection of genes for introduction 56, there are also risks of instability of introduced genes, possible loss of target genes after several generations, and a high rate of false positives 165. On the other hand, the CRISPR/CAS gene editing tool offers improved genetic stability by integrating target genes directly into the bacterial genome, facilitating the expression of target proteins 166. The bacterial genome can be edited to reduce toxicity 167, antigenicity, infectivity and increase the targeting colonizing ability of bacteria 168. Conventional physical and chemical methods, such as electro-transformation, also play a role in transfecting exogenous genes into probiotic cells 169.

**Figure 12:**
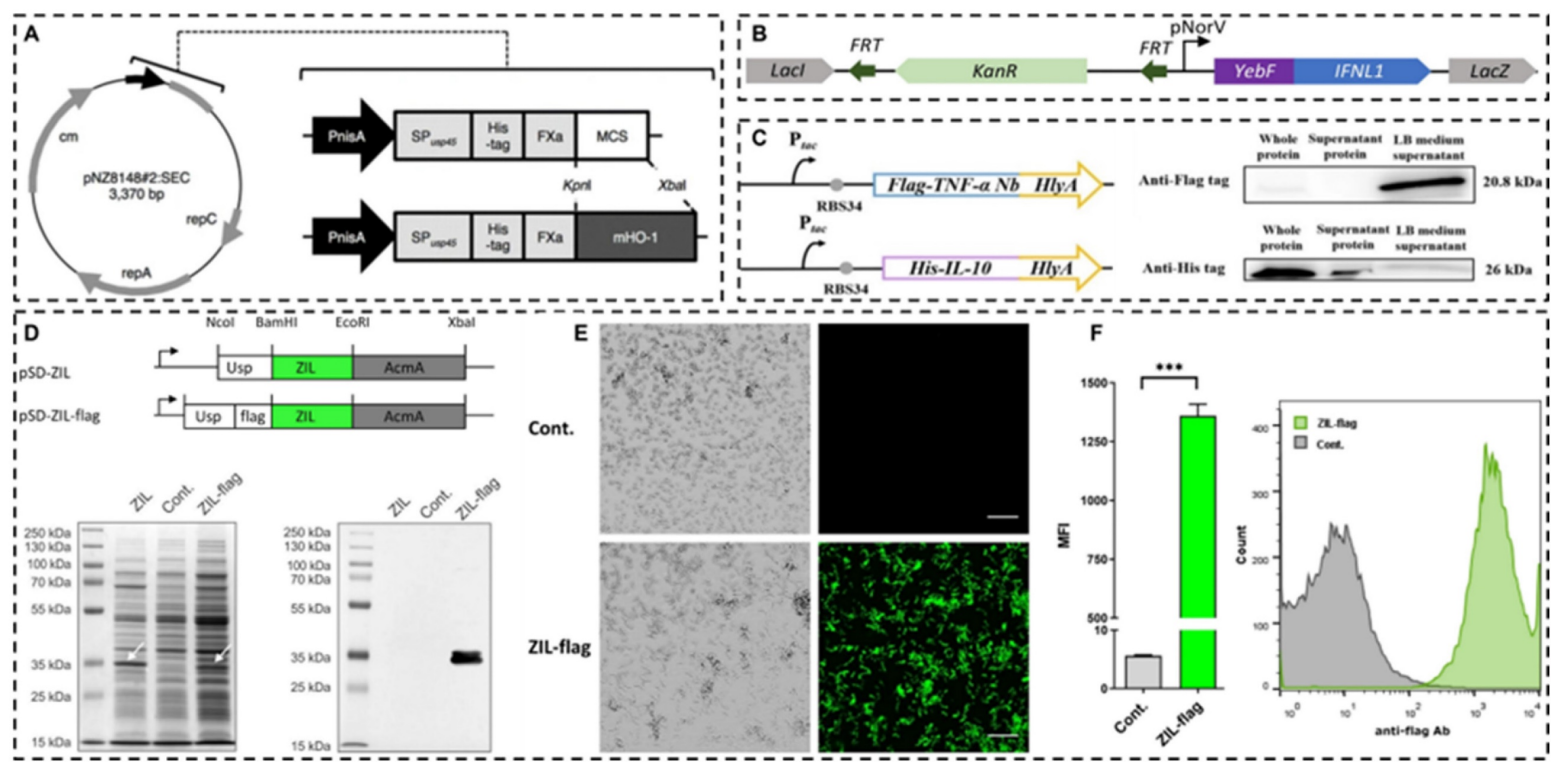
Recombinant probiotics for expressing anti-inflammatory mediators. **A**: A vector map of the *lactococcal* secretion vector and schematic representations of gene maps of the vector. Reproduced with permission from 173, copyright 2015, Springer Nature. **B**: Schematics of the IFN L1 production-secretion cassette for genomic integration. Reproduced with permission from 176, copyright 2023, America Chemical Society. **C**: Gene route design and Western blot detection results of constitutive engineered bacteria EcN-TNF-α Nanobodies (Nb, top) and EcN-IL10 (bottom). Reproduced with permission from 177, copyright 2024, Springer Nature. **D**: Gene constructs for expression of IL-6 binding affibody ZIL on the surface of *L. lactis* NZ9000, and SDS-PAGE, Western blot analysis of whole lysates. **E**: confocal immunofluorescence microscopy images. **F**: flow cytometry analysis. Reproduced with permission from 178, copyright 2022, Springer Nature.

The gene expression induction and regulation system of engineering bacteria developed in recent years can improve the expression efficiency of target genes by adding specific promoter sequences before target genes, including the chlorine induction system 170, lactic P170 induction system 171, phosphate induction system 172, peptide (nisin) regulatory expression system 171,173, sugar-induced expression systems, etc., which can switch the expression of target genes by adding various sugars to the diet of model animals *in vitro* 174,175. Shigemori *et al.* constructed a recombinant *Lactococcus lactis* NZ9000 capable of secreting the recombinant mouse heme oxygenase-1. The gene expression was controlled by the nisin-inducible promoter, and the plasmid was introduced into the strain using electroporation. Enzyme linked immunosorbent assay (ELISA) results showed that the cell extract of the strain contained a concentration of mouse heme oxygenase-1 of approximately 5μg/mL. Compared with the control strain, the recombinant strain could significantly increase the production of anti-inflammatory mediator IL-10 and reduce the expression of pro-inflammatory mediators in the colon 173. Chua *et al.* used *Escherichia coli* Nissle 1917 as the chassis, constructed an interferon λ1 (IFN L1) producing recombinant strain, and validated the therapeutic effect in the Caco-2/Jurkat T cell co-culture model and scaffold-based 3D co-cultured IBD model. The results showed that the engineered strains could protect the integrity of epithelial cells, increase TJ protein expression, reduce intestinal barrier permeability 176. Wu *et al.* constructed a dual bacterial system, the recombinant *Escherichia coli* Nissle 1917 can express and secrete anti-TNF-α nanobodies and IL-10, respectively, to simultaneously neutralize pro-inflammatory factors to enhance anti-inflammatory effects, regulate gut microbiota, and effectively inhibit colitis induced by DSS 177. Zahirović *et al.* constructed recombinant *Lactococcus lactis* NZ9000, which can express interleukin-6 (IL-6)-binding affibody on the surface of the strain to achieve IL-6 targeting function (**Figure 12**) 178. Comprehensive reviews on recent advancements in utilizing lactic acid bacteria as delivery vectors and related biological tools are available in the literature 161,179,180.

## 4. Clinical applications of probiotics for bowel disease

Current evidence suggests that probiotics may improve symptoms in patients with mild to moderate UC, though their efficacy in CD appears limited. It is important to acknowledge that existing clinical trial results are often inconsistent, and the lack of standardized experimental protocols contributes to this variability. Factors such as strain selection, dosage, treatment duration, as well as differences in patient characteristics and disease severity, complicate the ability to objectively evaluate the therapeutic potential of probiotics in IBD. Moreover, although both UC and CD fall under the category of IBD, significant differences exist in their underlying pathophysiology and clinical manifestations 25,26, which may also explain the differential responses to probiotic interventions.

Another crucial aspect of probiotic therapy is the potential risk of introducing pathogenic microorganisms. Given that IBD patients often exhibit an aberrant immune state within the intestines, and some may concurrently present with *Clostridium difficile* infections, the introduction of pathogenic bacteria could exacerbate the condition. Therefore, the choice of probiotic strains must take into consideration the patient's immune status, prioritizing strains with established safety profiles and suitable usage contexts. Even strains that are generally considered safe could have deleterious effects in immunocompromised individuals. Additionally, strict precautions should be taken during the production, transportation, storage, and administration of probiotics to prevent contamination with pathogenic microorganisms. Compliance with stringent quality control standards, along with proper transport and storage conditions, is essential, as is educating patients on appropriate usage.

At present, multiple probiotic strains have been approved for the treatment of IBD and have entered the clinical trial stage, including *Lactobacillus rhamnosus*, *Lactobacillus casei*, *Escherichia coli* Nissle 1917, VSL#3**^®^**, etc. Here we list the clinical trial records registered on the clinical trials website in **Table 2**. Considering the progress of clinical trials and the number of preclinical basic studies, we will briefly introduce VSL#3**^®^** and LGG**^®^** below.

### 4.1. VSL#3^®^

VSL#3^®^ (now branded as Visbiome^®^ in the U.S. and Vivomixx^®^ in Europe since January 31, 2016) is a commercially available probiotic blend comprising multiple probiotic strains 181, Visbiome^®^ is a mixed probiotic preparation consisting of eight live lyophilized probiotics, including four strains of *Lactobacillus* (*Lactobacillus casei, Lactobacillus plantarum, Lactobacillus acidophilus, and Lactobacillus delbrueckii*), three strains of *Bifidobacterium* (*Bifidobacterium longum, Bifidobacterium shortum,* and* Bifidobacterium infantis*), and one strain of *Streptococcus thermophilus*. These strains collectively exhibit the capacity to stably colonize the intestinal tract, reduce fecal pH value, locally augment FoxP3 expression in the intestine, and stimulate Treg cells, thereby modulating intestinal immunity upon sustained administration 182. It has been found that the IL10-induced CD4+ latency-associated peptide (LAP)+Treg pathway may be the main mechanism for its intestinal protective effect 183. A meta-analysis conducted in 2019 showed that Visbiome^®^ was significantly effective in relieving the symptoms of active UC without an increased risk of adverse effects 184. Moreover, in pediatric IBD patients, Visbiome® significantly outperformed placebo controls, reducing abdominal pain and rectal bleeding 185,186.

### 4.2. LGG^®^

LGG^®^ is another extensively investigated commercial probiotic product, comprising the *Lactobacillus rhamnoses* GG. This strain demonstrates robust tolerance to gastric and bile acids and has the capacity to secrete proteins that mitigate cytokine-induced apoptosis of epithelial cells 187,188. Evidence corroborates that a one-week administration of LGG^®^ facilitates colonization in the human colon 189. The secreted soluble protein HM0539 from LGG^®^ is documented to fortify intestinal integrity by modulating TJ protein expression and enhancing mucus secretion 190. Additionally, it attenuates Th17 cell activity, reduces IL-17 levels 191 and protects the intestinal epithelium through mucus-binding protein mediated bacteria-body interaction, displaying potent anti-inflammatory effects *in vitro* 192. LGG^®^-derived proteins P40 and P75 have been proven to be able to protect intestinal epithelial cells from apoptosis, promote proliferation, and activate Akt in a Phosphoinositide 3-Kinase-dependent manner 193. Specifically, P40 has been shown to enhance intestinal cell maturation and differentiation in newborn mice through the EGFR pathway 194. However, the results of several human clinical trials are conflicting and cannot confirm whether the therapeutic effect is superior to conventional 5-aminosalicylic acid therapy 195. This necessitates further clinical investigations to rigorously evaluate the therapeutic potential of LGG^®^ in IBD management.

## 5. Summary and outlook

**Figure 13:**
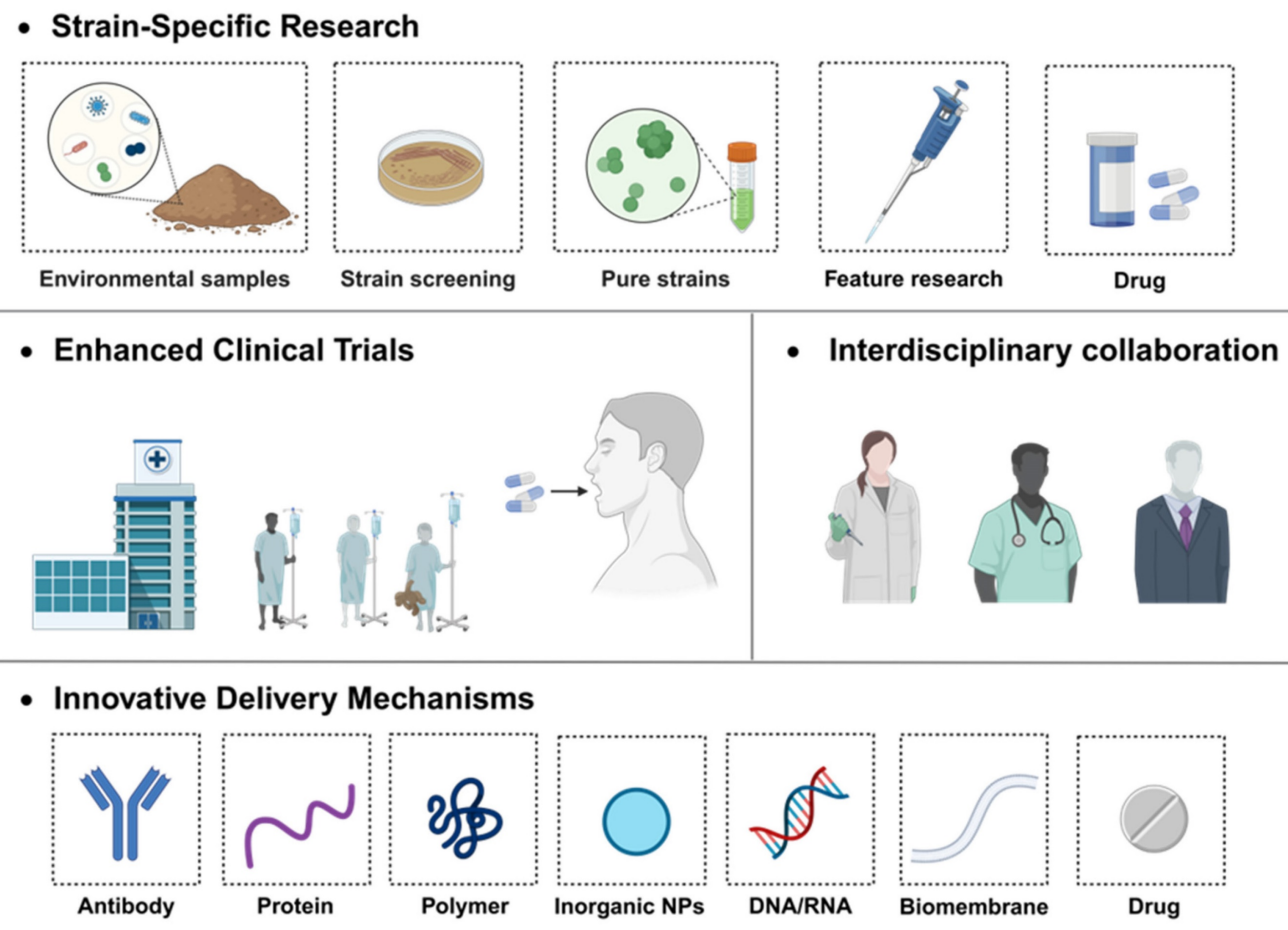
Schematic illustration of the future development direction of probiotics. Created with https://BioRender.com.

In this review, we have navigated through the intricate landscape of probiotic and their therapeutic implications in the realm of IBD. We extensively explored the diverse mechanisms by which probiotic exerts its influence, ranging from immunomodulation and antioxidant activities to fortifying the intestinal barriers and reshaping the gut microbiome. The narrative progressed to illuminate potential avenues for targeted therapy, spotlighting current innovations in bacterial modification techniques, and culminating with a critical appraisal of two leading probiotic products under extensive research scrutiny. Through promising, numerous hurdles persist within this domain, necessitating comprehensive and innovative solutions (**Figure 13**) to advance the field and enhance patient outcomes.

### 5.1. Strain-specific research

A deep dive into understanding the unique characteristics of different probiotic strains, ensuring a more tailored approach to their application in treating various forms of IBD. Various probiotics are playing an increasingly important role in the treatment of IBD due to their good safety and unique intestinal regulating function. However, the landscape is marred by the limited number of probiotic strains that have successfully transitioned into clinical trial phases with proven efficacy. The existing clinical studies, encompassing diverse patient demographics, geographical locations, and disease variants, further complicate the panorama due to variations in strain selection, dosage, and treatment duration. These factors, coupled with individual lifestyle choices and disease states, contribute to the considerable heterogeneity in clinical outcomes. As delineated by the 2023 ESPEN guideline on Clinical Nutrition in IBD, while probiotics show potential benefits for mild to moderate UC patients, their application in CD remains contentious and unsupported by robust clinical evidence 196.

### 5.2. Enhanced clinical trials with interdisciplinary collaboration

Rigorous, large-scale, multi-center clinical trials with standardized protocols should be conducted to validate the efficacy of specific strains in diverse patient populations. Looking ahead, the development of probiotic formulations comprising a consortium of strains holds promise for transcending the current limitations imposed by individual variability. This necessitates the initiation of meticulously designed, multi-center, and large-scale clinical trials, devoid of commercial bias, to unravel the intricate patient-probiotic interplay. In tandem, there is a pressing need for collaborative synergy across academia, clinical settings, and industry, aiming to translate scientific discoveries into tangible clinical solutions.

Advancing comprehensive therapies that incorporate bacterial metabolites and probiotic omics represents a promising approach to addressing the inconsistent clinical efficacy of probiotics. A substantial component of host-microbe interactions occurs through bacterial metabolites produced from the fermentation of intestinal contents. Metabolites such as short-chain fatty acids (SCFAs) exert significant regulatory effects on host physiology. Leveraging these metabolites, either as direct therapeutic agents or in conjunction with *in vivo* microbial interventions, may facilitate more consistent therapeutic outcomes. Furthermore, the development of personalized probiotic formulations, composed of diverse strains, their corresponding metabolites, and prebiotics tailored to the unique characteristics of individual patients, may yield superior clinical benefits compared to the use of single strains. Such combination therapies could lead to enhanced modulation of host microbiota and improved patient outcomes. Nevertheless, these approaches must undergo rigorous efficacy evaluation and be administered by healthcare professionals based on robust clinical evidence. Emerging research has demonstrated that these multi-component probiotic therapies exhibit enhanced efficacy in managing conditions such as inflammatory bowel disease and other gastrointestinal disorders. 197.

### 5.3. Innovative delivery mechanisms

Traditional chemical drugs and biological antibody therapies remain the primary treatment options for patients with IBD, with probiotics commonly used as adjunctive therapies. Existing research indicates that probiotics can offer therapeutic benefits, particularly in UC, where they have been shown to alleviate symptoms and reduce disease severity. Furthermore, probiotics generally exhibit fewer side effects and a higher safety profile, making them better tolerated by patients. When used in combination with conventional medications, probiotics can help mitigate drug-related side effects and potentially enhance overall treatment efficacy.

However, probiotics are not native to the human intestinal microbiota, and the gut environment is often not conducive to their colonization. To exert therapeutic effects, probiotics must withstand the harsh conditions of the digestive tract and reach the intestinal lumen. Additionally, individual variations in gut microbiota pose significant challenges to the consistent efficacy of probiotics. As a result, the impact of probiotics on treatment outcomes remains limited, and their use in conjunction with traditional therapies is currently a common strategy. Achieving an optimal balance between probiotics and pharmaceutical treatments is an area that warrants further investigation to maximize therapeutic benefits.

As we pivot to the product landscape, oral administration remains the predominant route for strain delivery. Nevertheless, this necessitates innovative approaches to enhance the strains' activity and colonization efficacy within the gastrointestinal milieu. Strategies such as prebiotic supplementation and advanced surface modification technologies have emerged, yet the field is ripe for further innovation, particularly in targeted modification methods and controlled release technologies, tailored specifically for IBD. Furthermore, ensuring prolonged strain viability outside laboratory settings remains a critical consideration for ensuring therapeutic efficacy upon patient administration.

In conclusion, as we grapple with the multifaceted effects of probiotics on human health, a paradigm shift towards a more diversified and nuanced evaluation of therapeutic efficacy is imperative. Future endeavors should be channeled towards establishing a robust classification system, capturing the spectrum of probiotics' effects across diverse patient populations. This, in turn, would facilitate more accurate and personalized evaluations, empowering healthcare practitioners, patients, and researchers alike, and ushering in a new era of optimized, patient-centric probiotic-based therapies.

## Figures and Tables

**Table 1:** Summary of probiotics for treating IBD.

Strains	Therapeutic agents	Model	Anti-inflammatory	Antioxident	Mucus/Goblet cells	Epithelial cells/TJs	SCFAs	Microbial regulation	Ref.
*L. acidophilus;L. helveticus;L. plantarum;*	strain	DSS-C57BL/6	+	+	+	+	+	+	30
*L. plantarum* 22A-3	strain	DSS-C57BL/6	+						31
*L. paracasei* BNCC345679	strain	DSS-C57BL/6	+	+		+		+	32
*L. plantarum* MKMB01/02	strain	HT-29, caco-2 and THP-1 cells	+		+	+			33
*L. acidophilus* LA1	strain	DSS-C57BL/6	+	+		+			34
*B. breve* FPHC4024;*L. reuteri* FPHC2951;	strain	DSS-C57BL/6J	+			+		+	35
*L. acidophilus* NCFM	Slp	LPS-RAW264.7 cells	+	+					36
*L. acidophilus* NCFM	Slp	DSS-C57BL/6;TNBS-C57BL/6	+						37
*A. muciniphila*	Slp	HFD-C57BL/6			+				38
*A. muciniphila*	Slp	CTL and CT26 Cells;DSS-C57BL/6J	+						39
*L. plantarum* AKU1009a	derivant	ICR	+	+					40
*L. plantarum* BMCM12	derivant	Type II mucin						+	41
*Lactobacillus plantarum* Q7	Extracellular vesicles	DSS-C57BL/6J	+		+	+		+	42
*L. lactis* NCDO 2118	strain	DSS-C57BL/6;Colonic cells	+			+			48
*L. casei* Shirota	strain	2,2′-Azobis (2-amidinopropane) dihydrochloride-Caco-2/TC7 cells	+	+		+			55
*L. plantarum* ZS62	strain	DSS-C57BL/6	+	+					58
*L. plantarum* FC225	strain	ICR		+					65
*B. bifidum*	strain	caco-2 cells;DSS-C57BL/6	+	+		+			75
*B. pseudocatenulatum*	strain	DSS-C57BL/6J	+		+	+		+	76
*B. bifidum*	strain	caco-2 cells;DSS-C57BL/6	+	+		+	+		77
*L. gasseri* ATCC33323	strain	DSS-C57BL/6	+			+		+	78
*L. acidophilus* CMUL067	strain	TNBS-BALB/c ByJ	+			+			79
*L. Plantarum*	Micro integral membrane protein	DSS-C57BL/6	+		+	+		+	80
*L. plantarum* HNU082	strain	DSS-C57BL/6	+		+	+	+	+	95
*L. casei;L. plantarum;L. rhamnosus;*	strain	HT-29 cells						+	109

**Table 2:** Clinical trials of probiotic for IBD therapies [Data from ClinicalTrials.gov, accessed on 6 June 2024.

NCT Number	Strain	Study Status	Conditions	Sponsor	Phases
NCT00114465	Mixed strains (VSL#3^®^)	COMPLETED	CD	Orphan Australia	PHASE4
NCT00175292	Mixed strains (VSL#3^®^)	COMPLETED	CD	University of Alberta	PHASE3
NCT00268164	*L. acidophilus* LA5; *B. animalis* BB12	TERMINATED	UC	Hvidovre University Hospital	PHASE2
NCT00367705	Mixed strains (VSL#3^®^)	UNKNOWN	UC	Hadassah Medical Organization	PHASE4
NCT00305409	*B. longum*	COMPLETED	CD	University of Dundee	NA
NCT00374374	*L. acidophilus;L. rhamnosus*	COMPLETED	CD	Odense University Hospital	NA
NCT00374725	*L. acidophilus;L. rhamnosus*	COMPLETED	UC	Odense University Hospital	NA
NCT00510978	*B. Infantis* 35624;	UNKNOWN	UC|CD	University College Cork	PHASE2/3
NCT00510978	*L. Salivarius* UCC118				
NCT00578799	Mixed strains (Kyo-Dophilus^®^)	WITHDRAWN	UC	University of California, Irvine	PHASE1
NCT00803829	Not Given	COMPLETED	UC	University of Dundee	NA
NCT00895336	*L. Rhamnosus* GG	WITHDRAWN	UC	Children's Hospital Medical Center, Cincinnati	PHASE2
NCT00944736	Mixed strains (VSL#3^®^)	COMPLETED	CD	Children's Mercy Hospital Kansas City	PHASE3
NCT00951548	Mixed strains (VSL#3^®^)	COMPLETED	UC	VSL Pharmaceuticals	NA
NCT01078935	Mixed strains	UNKNOWN	CD|UC	The Baruch Padeh Medical Center, Poriya	PHASE4
NCT01173588	Bifidobacterium	COMPLETED	IBD	Instituto Lala	PHASE3
NCT01193894	*L. plantarum* 299V	COMPLETED	UC	Nordisk Rebalance A/S	PHASE2|PHASE3
NCT01479660	Mixed strains (VSL#3^®^)	UNKNOWN	UC	Post Graduate Institute of Medical Education and Research, Chandigarh	PHASE4
NCT01548014	Mixed strains (VSL#3^®^)	UNKNOWN	CD	Samsung Medical Center	PHASE3
NCT01632462	Mixed strains (VSL#3^®^)	UNKNOWN	CD	Federico II University	PHASE4
NCT01698970	Not Given	COMPLETED	CD	Danone Research	NA
NCT01765439	Mixed strains (VSL#3^®^)	ACTIVE_NOT_RECRUITING	CD|UC	Charles University, Czech Republic	NA
NCT01765998	Not Given	UNKNOWN	CD	The Baruch Padeh Medical Center, Poriya	PHASE4
NCT01772615	*E. coli Nissle* 1917	COMPLETED	UC	Hvidovre University Hospital	PHASE4
NCT02361957	Mixed strains (Ecologic^®^825)	SUSPENDED	UC	Wageningen University	NA
NCT02488954	*P. freudenreichii*	TERMINATED	UC	Rennes University Hospital	NA
NCT03266484	Not Given	ACTIVE_NOT_RECRUITING	CD|UC	Massachusetts General Hospital	NA
NCT03415711	Mixed strains (VSL#3^®^)	TERMINATED	UC	VSL Pharmaceuticals	NA
NCT03798210	*L. reuteri* 4659	UNKNOWN	UC Flare	Uppsala University	PHASE2
NCT04006977	Mixed strains	UNKNOWN	UC	Xijing Hospital of Digestive Diseases	NA
NCT04102852	*L. Rhamnosus* GG	COMPLETED	UC|UC	San Giovanni Addolorata Hospital	PHASE1/2
NCT04223479	Not Given	COMPLETED	UC	University of Jordan	PHASE2/3
NCT04241029	Mixed strains (IDOFORM TRAVEL^®^)	COMPLETED	UC	Oslo University Hospital	NA
NCT04305535	Not Given	UNKNOWN	CD	Instituto de Investigación Sanitaria de la Fundación Jiménez Díaz	NA
NCT04804046	Not Given	TERMINATED	CD	University of Alberta	NA
NCT04842149	*B. Breve* Bif195	ACTIVE_NOT_RECRUITING	CD	Hvidovre University Hospital	NA
NCT04969679	*E. coli Nissle* 1917 (Mutaflor^®^)	COMPLETED	UC	Kangbuk Samsung Hospital	PHASE4
NCT05118919	*L. Reuteri* BGP-014	RECRUITING	UC	BioGaia Pharma AB	PHASE1/2
NCT05652621	Not Given	RECRUITING	UC|IBS	The First Affiliated Hospital of Xinxiang Medical College	NA
NCT05666960	Not Given	RECRUITING	UC	Rise Therapeutics LLC	PHASE1
NCT05906043	Not Given	RECRUITING	IBD	University College Dublin	NA
NCT06392061	Mixed strains (Trilac^®^)	RECRUITING	IBD	Lebanese University	NA
NCT06595719	Mixed strains	RECRUITING	UC	National University of Malaysia	NA
NCT06609447	Mixed strains (VSL#3^®^)	RECRUITING	UC	Second Affiliated Hospital, School of Medicine, Zhejiang University	PHASE4
NCT06637683	*B. subtilis*;*B. clausii* (LiveSpo COLON^®^)	COMPLETED	IBD	Anabio R&D	NA
NCT06642883	Mixed strains (ProLife^®^)	RECRUITING	UC	University of Padova	NA

## Abbreviations

IBD: inflammatory bowel disease
CD: Crohn's disease
UC: ulcerative colitis
TLR: toll-like receptor
Th cells: helper t cells
Treg cells: regulatory T cells
Slp: surface layer protein
DSS: dextran sulfate sodium
DCs: dendritic cells
IL-1β: interleukin-1β
IL-8: interleukin-8
ROS: reactive oxygen species
FPRs: formyl peptide receptors
CAT: catalase
SOD: superoxide dismutase
TNBS: trinitrobenzene sulfonic acid
NRF2: nuclear factor erythroid 2-related factor 2
NF-κB: nuclear factor kappa-B
ARE: antioxidant response element
GSH-Px: glutathione peroxidase
MAPK: mitogen-activated protein kinase
TJ: tight junction
MUC: mucins
SCFAs: short chain fatty acids
TNF-α: tumor necrosis factor-α
GPR43: g protein-coupled receptor 43
ZO-1: zonula occludens-1
HA: hyaluronic acid
SA: self-assemble
LBL: layer-by-layer
DBCO: dibenzocyclooctyne
CRISPR/CAS: clustered regularly interspaced short palindromic repeats/ crispr-associated systems
ELISA: Enzyme linked immunosorbent assay
IFN L1: interferon λ1
IL-6: interleukin-6
LAP: latency-associated peptide
ESPEN: the European Society for Clinical Nutrition and Metabolism
TEM: transmission electron microscopy
SEM: scanning electron microscope
UHR FE-SEM: ultra-high resolution field emission scanning electron microscope
